# Protein Transfer through an F Plasmid-Encoded Type IV Secretion System Suppresses the Mating-Induced SOS Response

**DOI:** 10.1128/mBio.01629-21

**Published:** 2021-07-13

**Authors:** Abu Amar M. Al Mamun, Kouhei Kishida, Peter J. Christie

**Affiliations:** a Department of Microbiology and Molecular Genetics, McGovern Medical School, Houston, Texas, USA; The Ohio State University

**Keywords:** SOS response, conjugation, type IV secretion, mutation, stress response, protein translocation

## Abstract

Bacterial type IV secretion systems (T4SSs) mediate the conjugative transfer of mobile genetic elements (MGEs) and their cargoes of antibiotic resistance and virulence genes. Here, we report that the pED208-encoded T4SS (Tra_pED208_) translocates not only this F plasmid but several plasmid-encoded proteins, including ParA, ParB1, single-stranded DNA-binding protein SSB, ParB2, PsiB, and PsiA, to recipient cells. Conjugative protein translocation through the Tra_pED208_ T4SS required engagement of the pED208 relaxosome with the TraD substrate receptor or coupling protein. T4SSs translocate MGEs as single-stranded DNA intermediates (T-strands), which triggers the SOS response in recipient cells. Transfer of pED208 deleted of *psiB* or *ssb*, which, respectively, encode the SOS inhibitor protein PsiB and single-stranded DNA-binding protein SSB, elicited a significantly stronger SOS response than pED208 or mutant plasmids deleted of *psiA*, *parA*, *parB1*, or *parB2*. Conversely, translocation of PsiB or SSB, but not PsiA, through the Tra_pED208_ T4SS suppressed the mating-induced SOS response. Our findings expand the repertoire of known substrates of conjugation systems to include proteins with functions associated with plasmid maintenance. Furthermore, for this and other F-encoded Tra systems, docking of the DNA substrate with the TraD receptor appears to serve as a critical activating signal for protein translocation. Finally, the observed effects of PsiB and SSB on suppression of the mating-induced SOS response establishes a novel biological function for conjugative protein translocation and suggests the potential for interbacterial protein translocation to manifest in diverse outcomes influencing bacterial communication, physiology, and evolution.

## INTRODUCTION

The type IV secretion systems (T4SSs) translocate two main types of macromolecules, DNA and proteins, to bacterial or eukaryotic target cells (1, 2). Members of one large subfamily, the conjugation systems, deliver mobile genetic elements (MGEs) to recipient bacteria, while those of a second, the effector translocators, transmit protein substrates to eukaryotic cells to aid in infection processes (1, 3). Based on detailed phylogenetic analyses of conserved components of T4SSs, it has been proposed that conjugation systems arose first in diderms (Gram-negative species), adapted for DNA transfer in monoderms (Gram-positive species), and most recently, functionally diversified as effector translocators during establishment of bacterial pathogenic or symbiotic relationships with eukaryotes (4, 5). The T4SS subfamily of conjugation systems is unique among the known bacterial secretion systems in the capacity to deliver DNA substrates intercellularly (6), which raises the intriguing questions of how conjugation machines evolved in the first place and how they were then reconfigured as protein translocators. A key mechanistic feature of conjugation, namely, that the DNA transfer intermediate consists of a single strand of DNA covalently bound at its 5′ end by a protein termed the relaxase (7), illuminates an understanding of both the genesis of conjugation systems and their eventual exaptation as effector translocators.

During conjugation, two distinct sets of proteins spatially and temporally coordinate their activities to process and transfer DNA substrates across the donor cell envelope (7). The DNA replication and transfer (Dtr) proteins process DNA destined for transfer by assembling at origin-of-transfer (*oriT*) sequences harbored by MGEs. One Dtr subunit, the relaxase, nicks the DNA strand destined for transfer (the T-strand) in a phosphodiesterase cleavage reaction that covalently tethers the relaxase to the 5′ end of the T-strand. The transfer (Tra) proteins assemble as the T4SS channel, and one component of the channel termed the VirD4 substrate receptor recruits the relaxosome through recognition of translocation signals (TSs) carried by the relaxase and other Dtr factors (8–11). The VirD4 receptor (also termed the type IV coupling protein or T4CP) coordinates further processing and delivery of the relaxase−T-strand nucleoprotein particle (the T-complex) in a 5′-to-3′ direction through the T4SS channel (7, 12). Most relaxases resemble rolling-circle replicases in structure and enzymatic action based on a common HUH (His-hydrophobic-His) motif in the catalytic pocket (13). Relaxases confer recognition of associated DNA as a substrate, and they also “pilot” covalently tethered T-strands to target cells. There is also evidence that relaxases can translocate intercellularly independently of their DNA substrates (14–18). Together, these findings support a general model that conjugation systems arose through the capacity of ancestral protein translocation systems to recognize rolling-circle replicases as secretion substrates. Then, with the emergence of eukaryotic cells, conjugation systems evolved as effector translocators through adaptations in the VirD4 receptor that enabled recruitment of distinct protein repertoires to the translocation or “mating” channel (4). Indeed, the notion that VirD4 receptors regulate protein substrate flow through T4SS channels is supported by several recent findings, including evidence that chimeric VirD4 receptors can be engineered to translocate nonnative protein substrates interbacterially (19) and structural definition of the effector-VirD4 receptor interface in the Legionella pneumophila Dot/Icm system (20–22).

In this study, we tested an overarching hypothesis that “dedicated” conjugation machines naturally translocate a larger repertoire of protein substrates to other bacteria than previously envisioned. We report that the IncFV plasmid pED208 conjugatively transfers the TraI relaxase as well as six other plasmid-encoded proteins whose functions are associated with plasmid maintenance during vertical or horizontal transmission. We define the genetic requirements for protein transfer and present evidence that translocation of two proteins, PsiB and single-stranded DNA (ssDNA)-binding protein SSB, suppresses the SOS response, which is activated in recipient cells upon receipt of the incoming ssDNA transfer intermediate. We thus identify a novel biological function for conjugative protein translocation of proposed importance for long-term plasmid survival and genome evolution.

## RESULTS

### Plasmid maintenance proteins are translocated through the Tra_pED208_ T4SS.

We selected six candidate substrates with functions predicted to promote establishment of the newly transferred F plasmid in recipient cells. The IncFV plasmid pED208 served as our model F plasmid due to the fact that it elaborates many T4SS channels on the cell surface and efficiently transfers between cells (23, 24). pED208’s transfer region was sequenced previously (23, 25), and recently, we completed the sequence of the entire plasmid (A. A. M. Al Mamun et al., unpublished data). The candidate substrates included the pED208-encoded partitioning proteins ParA and ParB, which share 21.5 and 23.1% identities, respectively, with SopA and SopB of the classical F plasmid. These substrates were selected because of recent evidence that the partitioning protein, ParM, encoded by the IncFII plasmid R1-16 is conjugatively transferred to recipient cells (26). Other candidate substrates were encoded by a cluster of genes, *ssb-parB2-psiB-psiA*, that have been shown to be broadly conserved among large conjugative plasmids of the IncF, IncI, and many other incompatibility groups (27, 28). These proteins are predicted to function in plasmid maintenance, and PsiB also has been shown to suppress the SOS response, a stress response that is activated in recipient cells upon acquisition of the incoming ssDNA transfer intermediate (28–31).

**FIG 1:**
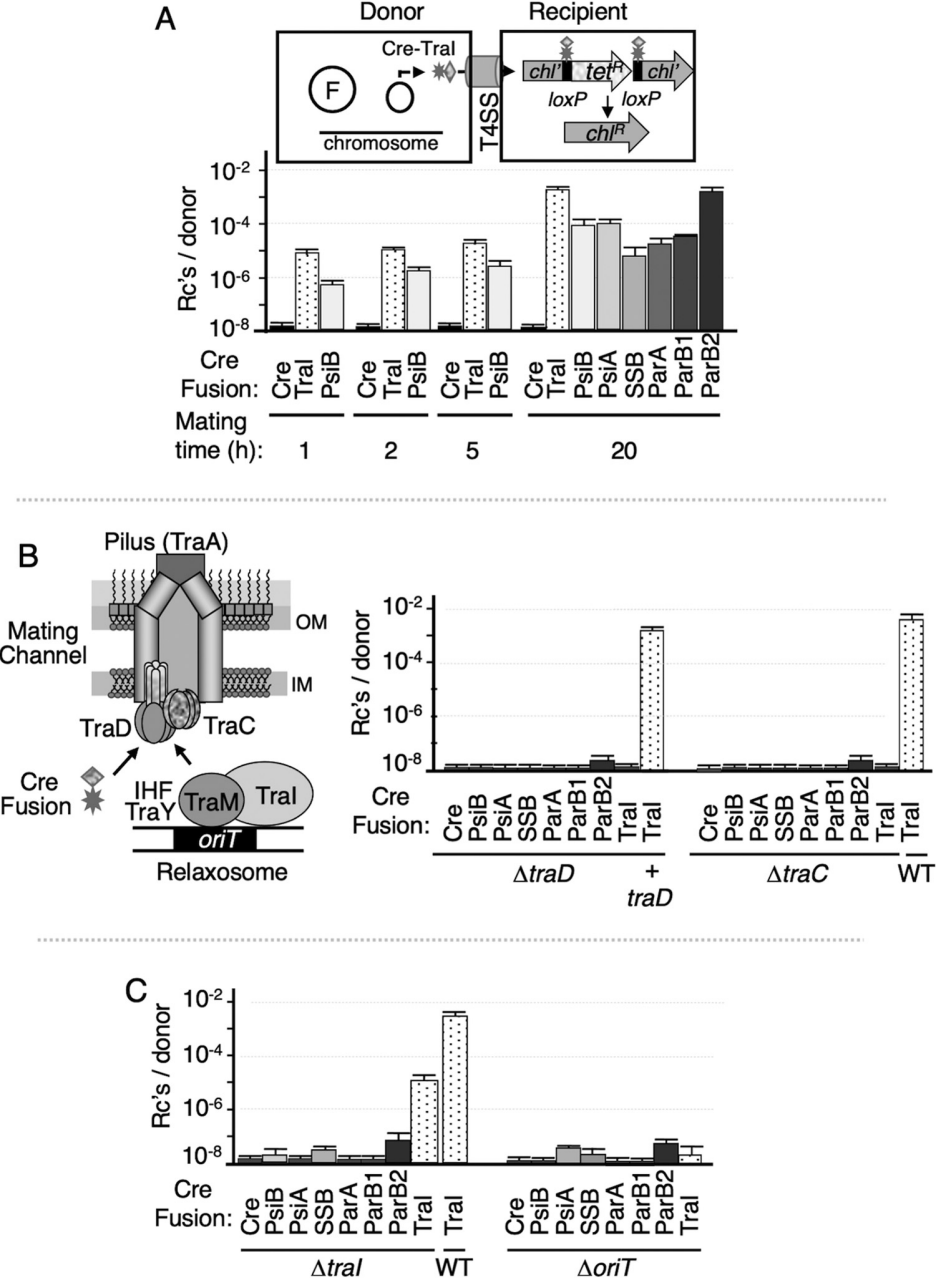
Conjugative transfer of pED208-encoded plasmid maintenance proteins through the Tra_pED208_ T4SS. (A) Schematic of CRAfT. Donor strains carry pED208 or mutant derivatives (designated F) encoding the Tra_pED208_ T4SS and a nontransmissible plasmid producing the Cre fusion protein of interest. Translocation of the Cre fusion protein results in excision of the *lox* cassette and conversion of recombinant cells from Chl^s^ Tet^r^ to Chl^r^ Tet^s^. (Bottom) Transmission of Cre only (as a negative control), Cre-TraI (as a positive control), or the Cre-fused maintenance proteins at the times indicated. The number of recombinants arising from *lox* excision per donor cell (Rc’s/D) is shown on the *y* axis. (B) Simplified schematic of Tra_pED208_ T4SS highlighting the TraA pilin, VirD4-like TraD substrate receptor, and VirB4-like TraC ATPase. The schematic also shows the pED208 relaxosome highlighting the TraI relaxase, TraM Dtr (DNA replication and transfer) factor, and *oriT* sequence. OM, outer membrane; IM, inner membrane. (Right) Effects of Δ*traD* and Δ*traC* mutations on translocation of Cre fusion proteins indicated. WT, Cre-TraI transfer by the pED208 donor was used as a positive control. (C) Effects of Δ*traI* and Δ*oriT* mutations on translocation of Cre fusion proteins indicated. WT, transfer of Cre-TraI by the pED208 donor. All transfer experiments were repeated at least three times in triplicate. Results are reported as the mean frequency of transfer with the standard error of the mean (error bar).

We assayed for conjugative protein translocation by use of the Cre recombinase assay for translocation (CRAfT), an assay widely deployed to identify candidate substrates of effector translocator T4SSs (15, 32–34). Candidate substrates were fused to the Cre recombinase, and the fusion proteins were tested for transfer from donors harboring pED208 to a reporter recipient bearing a *lox* cassette in the chromosome (Fig. 1A). Relaxases have been shown by CRAfT and other assays to be translocated through conjugation systems (15, 16, 34, 35). In agreement with those findings, pED208-carrying donors efficiently translocated Cre fused to the pED208-encoded TraI relaxase, but not Cre alone (Fig. 1A). Cre-TraI translocation occurred at a frequency of ∼10^−5^ *lox* recombinants per donor (Rcs/D) within 1 h of the onset of mating and at higher frequencies with longer mating times, ultimately reaching ∼10^−2^ Rcs/D in 20-h matings, which is typically the duration used for CRAfT. Remarkably, pED208 donors also translocated Cre when fused to each of the six maintenance proteins under study (Fig. 1A). pED208-carrying donors translocated Cre-PsiB at frequencies comparable to those of Cre-TraI at all tested mating times. In 20-h matings, all six Cre fusion proteins were translocated at frequencies between 10^−3^ Rcs/D for Cre-ParB2 and ∼10^−6^ Rcs/D for Cre-SSB. Donors deleted of essential subunits, including the VirD4-like receptor TraD or the VirB4-like ATPase TraC failed to translocate the Cre fusion proteins (Fig. 1B), confirming that an intact pED208-encoded Tra T4SS (termed Tra_pED208_ T4SS) is required for intercellular transfer of these protein substrates.

### Translocation of maintenance proteins requires assembly of the pED208 relaxosome and its engagement with the TraD substrate receptor.

We extended these initial findings in two directions, first, by defining the genetic requirements for conjugative protein translocation, and second, by assaying for biological functions of the translocated proteins in recipient cells. For conjugative DNA transfer, the Dtr proteins must assemble at the *oriT* sequence to form the catalytically active relaxosome (7). There is accumulating evidence that binding of relaxosomes with VirD4-like substrate receptors serves as an activating signal for DNA substrate processing and translocation. On the one hand, this contact stimulates processing of the DNA substrate, while on the other hand, it activates the receptor by stimulating oligomerization and ATP hydrolysis activity as well as productive coupling with the cognate T4SS channel (18, 36–42). In earlier studies, Lang, Zechner, and colleagues supplied evidence that the relaxosome of R1-16 must engage with TraD for translocation of Cre-TraI as well as heterologous DNA substrates such as the mobilizable plasmids ColE1 and CloDF13 (16, 43). To test whether relaxosome-TraD coupling is a general requirement for protein trafficking through an F system, we constructed and analyzed the effects of *dtr* gene and *oriT* deletions on substrate trafficking through the T4SS_pED208_. In F plasmids, the Dtr processing proteins include the plasmid-encoded TraM and TraY accessory factors and TraI relaxase, and host-encoded integration host factor (IHF) (7, 44). The assembled F relaxosome interacts with the TraD receptor via unspecified contacts involving TraI’s internal translocation signals (translocation signal A [TSA] and B [TSB]) (10) and a structurally defined interaction between TraM’s C-terminal tetramerization domain and the last ∼15 residues of TraD (8, 45).

In line with previous findings (8, 43, 46), the Δ*oriT* and Δ*traI* mutations completely blocked plasmid transfer, while deletions of *traM* and codons for the last 15 residues of TraD that bind TraM (*traD*Δ*C15*) conferred attenuated transfer by ≥2 orders of magnitude (see Fig. S1A in the supplemental material). The Δ*traI*, Δ*traM*, Δ*oriT*, and *traD*Δ*C15* mutations had no detectable effects on production of the pED208 F pilus or M13 phage infection (Fig. S1B). The Δ*traI* and Δ*traM* mutations were complemented by *trans*-expression of the corresponding genes, establishing that the deletions did not exert polar effects on expression of the other *tra* genes (Fig. S1A). pED208Δ*oriT* also mobilized the transfer of a p*oriT* plasmid harboring pED208’s *oriT* sequence, confirming that the Δ*oriT* mutation does not block expression of pED208’s *tra* functions (Fig. S1A).

pED208Δ*traI*-carrying donors delivered Cre-TraI to recipients, albeit at frequencies of ∼2 orders of magnitude lower than those observed for pED208-carrying donors (Fig. 1C). Notably, all tested recombinants (1,000 of 1,000) that were recovered from Cre-TraI transfer also carried the pED208Δ*traI* plasmid. Cre-TraI thus retains the capacity to process and pilot the DNA transfer intermediate to recipient cells, albeit at reduced frequencies that likely can be attributed to steric effects of the Cre moiety on relaxase processing or piloting functions. Escherichia coli MC4100(pED208Δ*oriT*) donors were abrogated for Cre-TraI transfer, consistent with previous findings for the R1-16 system (Fig. 1C) (35, 43). Donors harboring the pED208Δ*traI* or pED208Δ*oriT* mutant plasmids also were blocked for transfer of the Cre-maintenance fusion proteins (Fig. 1C). This result is significant because, in contrast to TraI, these proteins are not predicted to associate covalently or even noncovalently with the T-strand during translocation. pED208 relaxosome assembly and engagement with the TraD receptor thus appears to be a general requirement for conjugative translocation of these and possibly other protein substrates through the Tra_pED208_ T4SS.

10.1128/mBio.01629-21.5 FIG S1 Phenotypes of pED208 mutations. Download FIG S1, PDF file, 1.9 MB. Copyright © 2021 Al Mamun et al. 2021 Al Mamun et al. https://creativecommons.org/licenses/by/4.0/ This content is distributed under the terms of the Creative Commons Attribution 4.0 International license.

**FIG 2:**
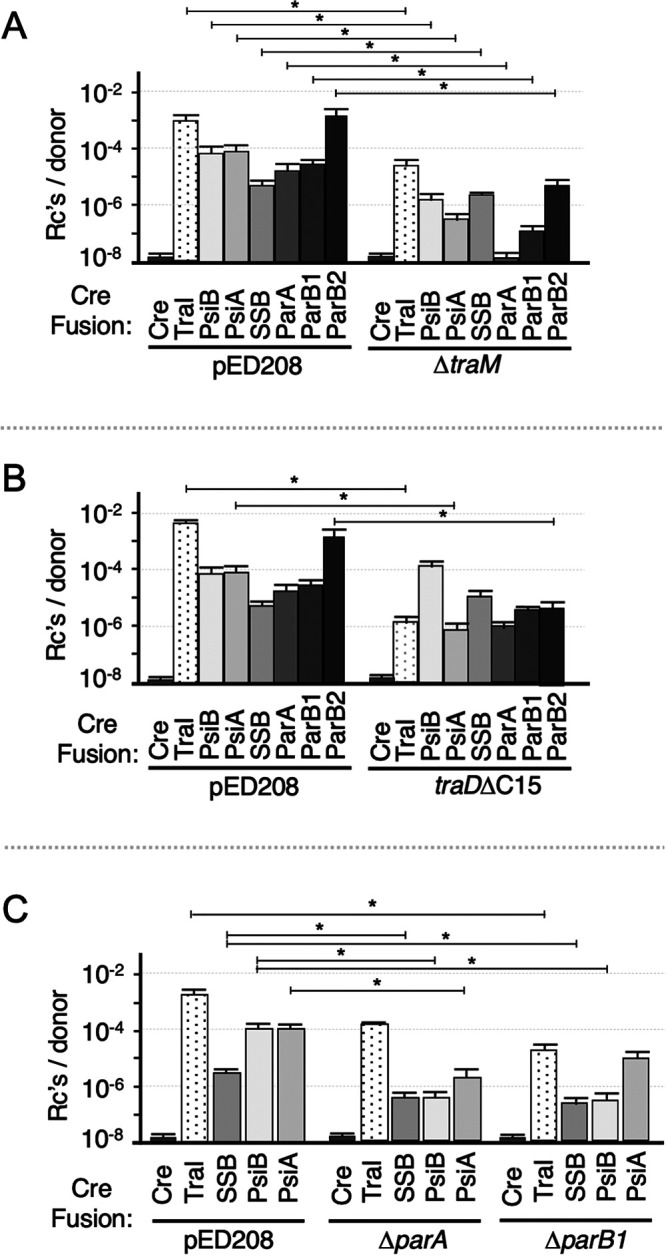
Contributions of the Dtr (DNA replication and transfer) factor TraM, the TraM-interacting domain of the TraD receptor, and partitioning proteins ParA and ParB1 to translocation of Cre fusion proteins. (A to C) Transfer of Cre only, Cre-TraI, or the Cre-fused maintenance proteins by donors harboring pED208 compared with pED208Δ*traM* (A), pED208traDΔ*C15* (B), or pED208Δ*parA* or pED208Δ*parB1* (C). Transfer experiments were repeated at least three times in triplicate. Results are reported as the mean frequency of transfer with the standard error of the mean. *P* values were determined by two-tailed Student’s *t* test for transfer frequency of Cre-fusion proteins by pED208 variants indicated compared in parallel with that of the respective Cre-fusion proteins by the WT pED208 donor in the same experiments. *P* values are shown above the bars as follows: *, *P* ≤ 0.05.

TraM promotes relaxosome assembly through binding of its N-terminal ribbon-helix-helix (RHH) domain to *sbm* sites in the *oriT* sequence and a presumptive C-terminal contact with TraI (38, 47–49). As noted above, TraM’s C terminus also forms a specific contact with TraD to promote coupling of the DNA substrate with the receptor/T4SS channel complex (8). In other F plasmids, TraM also positively regulates expression of the *tra* genes, which complicates assessments of TraM’s role in coordinating substrate trafficking through cognate T4SSs (43, 50). In pED208, however, an insertion sequence (IS) element with an outward-reading promoter at the 5′ end of the *tra* operon confers constitutive expression of the *tra* operon (23). Accordingly, pED208 elaborates abundant Tra_pED208_ T4SSs independently of TraM transcriptional control (see Fig. S1), which enabled us to evaluate TraM’s contributions to protein trafficking. Interestingly, pED208Δ*traM* donor strains were attenuated for translocation of Cre-TraI as well as each of the Cre-maintenance protein fusions; most notably, translocation of Cre-ParA was completely blocked (Fig. 2A). The absence of TraM might compromise relaxosome assembly, relaxosome-TraD docking, or both. To examine the role of the TraM-TraD interaction to protein trafficking, pED208Δ*traD* mutant donors were engineered to produce TraDΔC15 (8). Deletion of the C-terminal residues correlated with reductions in the translocation efficiencies of all Cre fusion proteins, although at levels that were statistically significant only for Cre-TraI, Cre-PsiA, and Cre-ParB2 (Fig. 2B). Overall, the data support a general stimulatory effect of the TraM-TraD interaction on protein trafficking.

We and others have reported that partitioning proteins also stimulate, or are essential for, translocation of DNA substrates through other T4SSs (26, 51–53). Where characterized, the partitioning proteins form interaction networks with Dtr components of the relaxosome and the VirD4 substrate receptor, suggesting that Par proteins act by promoting the coupling of relaxosomes with cognate T4SSs (26, 51). To determine whether partitioning proteins play similar roles in stimulating protein trafficking through the Tra_pED208_ T4SS, we deleted *parA* and *parB1*. Initial phenotypic studies confirmed that the Δ*parA* and Δ*parB1* mutations had no discernible effects on elaboration of pED208 F pili as assessed by: (i) a pilus-mediated aggregation assay, (ii) detection of TraA pilin in the culture supernatant, and (iii) susceptibility to infection by the bacteriophage M13, which binds the F pilus to gain entry into the bacterial host (Fig. S1B). These *par* mutations also did not detectably impact plasmid transfer efficiencies in liquid matings in durations of 5 to 90 min (Fig. S1C). However, the Δ*par* mutant donors translocated Cre-TraI, Cre-SSB, Cre-PsiB, and Cre-PsiA at diminished frequencies compared with wild-type (WT) pED208 donors (Fig. 2C), suggesting that pED208-encoded ParA and ParB1 stimulate protein, albeit not plasmid, transfer.

### pED208 transfer elicits the SOS response, and *psiB* and *ssb* mutations significantly enhance SOS induction.

To assay for biological activities associated with protein translocation, we constructed pED208 variants deleted of *ssb*, *parB2*, *psiB*, or *psiA*. Reminiscent of the Δ*parA* and Δ*parB1* mutations, these deletions did not detectably impact assembly of the Tra_pED208_ T4SS as monitored by F pilus production (Fig. S1B) and plasmid transfer over a range of mating times (Fig. S1C). Unlike the effects of Δ*parA* and Δ*parB1* mutations on protein trafficking, however, deletions of *ssb* or *parB2* had no effects on translocation of Cre-TraI or Cre-PsiB. The *psi* (plasmid SOS inhibition) locus originally identified on F and other conjugative plasmids was named for its ability to block the temperature-inducible SOS response of a *recA441* mutant, as evidenced by inhibition of SOS-dependent prophage λ induction and *sfiA* expression (27, 28, 54). The *psi* locus carries *psiB* and *psiA*, but only *psiB* expression was found to inhibit the SOS responses of strains bearing *recA441* or other SOS-activating mutations (29, 54). The findings, and evidence that mobile genetic elements (MGEs) are translocated intercellularly as ssDNA intermediates (55), led to the proposal that large conjugative plasmids carry *psiB* genes to suppress the SOS response induced during mating (29, 54). Such a function was envisioned to protect new transconjugants from the potentially deleterious consequences of SOS-enhanced mutation and recombination (54, 56).

**FIG 3:**
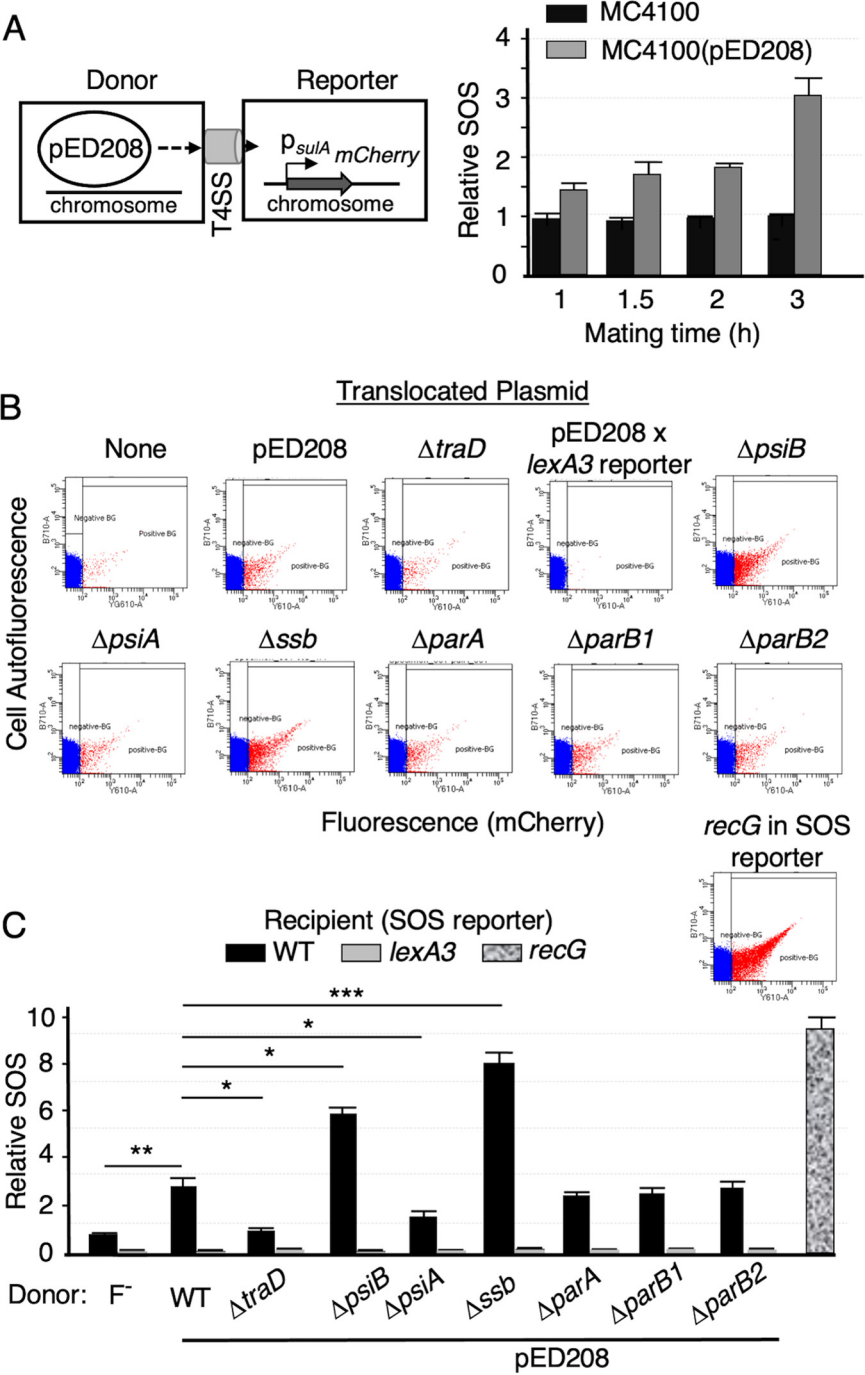
Effects of conjugative transfer of wild-type or mutant pED208 plasmids on the SOS response in recipient cells. (A) Schematic showing the E. coli MC4100(pED208) donor mated with the plasmid-free SOS reporter strain. (Right) SOS responses elicited by matings with MC4100 or MC4100(pED208) as a function of mating time, as determined by flow cytometry. Results are presented as the relative SOS response, which corresponds to the ratio between the numbers of cells exhibiting SOS induction in the experimental mating versus a mating with the plasmid-free donor (see Materials and Methods). (B) Representative examples of flow cytometry data. Data points indicate flow cytometry events (cells) colored red to the right (SOS induced) and blue to the left (SOS uninduced) of the “red” gate, which was set using the SOS-uninducible *lexA3* mutant strain. Panels depict flow cytometry data for donors harboring pED208 or the mutant plasmids listed. As a negative control and to set the “red” gate, MC4100(pED208) was mated with the isogenic reporter strain harboring the *lexA3* allele, which blocks SOS induction. As a positive control for SOS induction, the SOS response of the reporter strain harboring *recG*, which confers a high-level SOS response, was quantitated. None, mixture of plasmid-free MC4100 with the SOS reporter strain. (C) Quantitation of the relative SOS responses elicited by matings between donors harboring the pED208 variants shown and the WT or *lexA3* SOS reporter strains. SOS response of the SOS-constitutive *recG* mutation in SOS reporter strain is depicted at the right. (see also Fig. S2A and Table S3 in the supplemental material). Values are means plus standard errors of means (SEM) (error bars). *P* values are shown above the bars as follows: *, *P* ≤ 0.05; **, *P* ≤ 0.005; ***, *P* ≤ 0.0005.

To test whether pED208-encoded PsiB blocks the mating-induced SOS response, we employed a flow cytometry assay developed to monitor effects of DNA damage agents on SOS induction in single cells (57). Mixtures of E. coli MC4100(pED208) donors or plasmid-free MC4100 and an SOS reporter strain bearing P*_sulA_-mCherry* in its chromosome were monitored for changes in the numbers of cells exhibiting mCherry fluorescence by flow cytometry (58) (Fig. 3A). Within 1 h of mixing, MC4100(pED208) elicited a higher SOS response than MC4100, as evidenced by an increase in the numbers of SOS reporter cells exhibiting *mCherry* fluorescence at levels above a “red” gate, which was set with the SOS induction-deficient *lexA3* reporter strain (Fig. 3A and B). The relative SOS response triggered by MC4100(pED208) increased with longer mating times of 1.5 and 2 h, and at 3 h, it was ∼3-fold higher than that triggered by MC4100 (Fig. 3A). The kinetics of SOS induction observed by flow cytometry is in general agreement with results obtained in matings between an E. coli Hfr donor and E. coli or Salmonella enterica serotype Typhimurium strains carrying the *recA*::*lacZ* reporter (30). Importantly, the pED208Δ*traD*-carrying donor strain triggered an SOS response comparable to that of plasmid-free MC4100, confirming that ssDNA transfer is required for SOS induction (Fig. 3B and C). Furthermore, the MC4100(pED208) donor failed to induce P*_sulA_-mCherry* expression in the *lexA3* reporter strain (Fig. 3), establishing that conjugative DNA transfer induces P*_sulA_-mCherry* expression through activation of the SOS response. Finally, we compared the mating-induced SOS response with that of an SOS reporter bearing a *recG* mutation, which confers high basal expression levels of cellular SOS genes (59, 60). Although MC4100(pED208) donors activated the SOS response in only ∼0.2% of the recipient cells (Fig. 3B and C; see also Table S3 in the supplemental material), this level of SOS induction was within an order of magnitude of that observed with the SOS-constitutive *recG* reporter strain (see Discussion) (57, 58, 61).

10.1128/mBio.01629-21.3 TABLE S3 SOS induction during mating. Download Table S3, PDF file, 0.01 MB. Copyright © 2021 Al Mamun et al. 2021 Al Mamun et al. https://creativecommons.org/licenses/by/4.0/ This content is distributed under the terms of the Creative Commons Attribution 4.0 International license.

10.1128/mBio.01629-21.6 FIG S2 SOS responses elicited by pED208 variants in the *lexA3* SOS reporter strain. Download FIG S2, PDF file, 0.2 MB. Copyright © 2021 Al Mamun et al. 2021 Al Mamun et al. https://creativecommons.org/licenses/by/4.0/ This content is distributed under the terms of the Creative Commons Attribution 4.0 International license.

Strikingly, delivery of pED208Δ*psiB* into the SOS reporter triggered a significantly stronger SOS response than transfer of WT pED208 (Fig. 3B and C, Fig. S2A, and Table S3). To determine whether other maintenance genes modulate the SOS response, donors harboring the other pED208 variants were mated with the SOS reporter. Translocation of pED208Δ*ssb* stimulated an even stronger SOS response than pED208 or the Δ*psiB* mutant plasmid, whereas pED208 variants deleted of *parA*, *parB1*, *parB2*, or *psiA* failed to induce SOS responses over levels observed with pED208 transfer (Fig. 3B and C, Fig. S2A, and Table S3). In fact, translocation of pED208Δ*psiA* resulted in a statistically significant reduction in the SOS response, suggesting that PsiA might counteract the suppressive effects of PsiB or SSB on SOS induction. Together, these findings firmly established that translocation of the pED208 transfer intermediate induces the SOS response and that PsiB or SSB production in donor or recipient cells suppresses this response.

### Translocation of PsiB or SSB, but not PsiA, through the Tra_pED208_ T4SS suppresses the SOS response.

**FIG 4:**
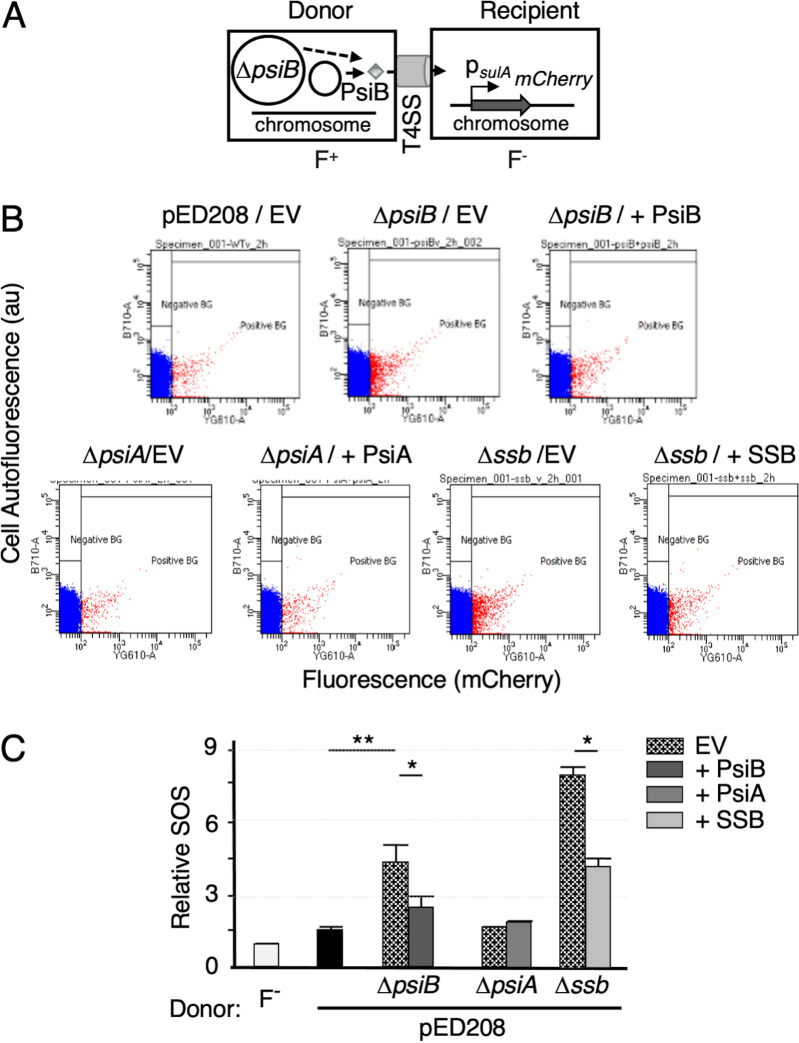
Effects of PsiB, PsiA, or SSB translocation on the SOS response. (A) Schematic shows MC4100 harboring transmissible pED208Δ*psiB* and a nontransmissible plasmid expressing *psiB* mated with the SOS reporter. (B) Representative examples of flow cytometry data showing effects of translocated PsiB, PsiA, or SSB on the SOS response in the recipient reporter strain. Panels depict flow cytometry data (in cell autofluorescence as arbitrary units [au]), as described in the Fig. 3 legend, for donors harboring pED208 or mutant plasmids listed with nontransmissible expression plasmids producing the complementing proteins or empty vector (EV). BG, background; positive, fluorescence above *lexA3* mutant; negative, fluorescence less than *lexA2* mutant. (C) Quantitation of the relative SOS responses elicited by strains presented in panel B (see also Table S4 in the supplemental material). *P* values were determined by two-tailed Student’s *t* test for SOS induction for transfer of pED208 variants bearing the respective clones in pBAD24 compared in parallel with that of pED208 variants bearing empty vector (EV) strains in the same experiments. *P* values are shown above the bars as follows: *, *P* ≤ 0.05; **, *P* ≤ 0.005.

To test whether translocation of PsiB or SSB through the Tra_pED208_ T4SS suppresses the mating-induced SOS response, we engineered donor strains to carry the transmissible plasmids pED208Δ*psiB* or pED208Δ*ssb*, plus plasmids expressing *psiB* or *ssb* (Fig. 4A). Initial studies confirmed that these expression plasmids are not mobilized at detectable frequencies by the pED208-encoded T4SS. PsiB or SSB produced in donors suppressed the strong SOS responses accompanying transfer of the Δ*psiB* or Δ*ssb* mutant plasmids in recipient cells (*P* ≤ 0.05) (Fig. 4B and C, Fig. S2A, and Table S3). We also tested for but did not detect modulation of the mating-induced SOS response when donors harbored pED208Δ*psiA* and *psiA* expressed from a nontransmissible plasmid (Fig. 4B and C, Fig. S2A, and Table S3). We conclude that translocation of PsiB or SSB, but not PsiA, through the Tra_pED208_ T4SS suppresses the mating-induced SOS response in recipient cells.

10.1128/mBio.01629-21.4 TABLE S4 SOS activation in plasmid-carrying populations. Download Table S4, PDF file, 0.01 MB. Copyright © 2021 Al Mamun et al. 2021 Al Mamun et al. https://creativecommons.org/licenses/by/4.0/ This content is distributed under the terms of the Creative Commons Attribution 4.0 International license.

The above findings were obtained by *trans*-expressing *psiB*, *ssb*, and *psiA* from a multicopy plasmid. To assess biological relevance, we asked whether pED208-carrying donors express the SOS suppressing genes from their native loci. We first quantitated expression of *psiB*, *ssb*, and *psiA* in donor cells by real-time reverse transcription-PCR (RT-PCR) and determined that all three genes were expressed at 1.8-fold or higher levels compared to a *gyrA* housekeeping gene (Fig. S3A) (62). Next, we incorporated a streptactin epitope tag at the native *ssb* locus and assayed for protein production. SSB-*str* was readily detected in pED208::*ssb-str*-carrying donors and, interestingly, accumulated at ∼1.3-fold-higher levels in 3-h mating mixes (Fig. S3B). pED208-carrying donors thus express *ssb* and *psiB* and synthesize SSB protein. The elevated accumulation of Str-SSB in mating mixes compared with donor-only populations might derive from stimulated gene expression in donors or new transconjugants (see Discussion).

10.1128/mBio.01629-21.7 FIG S3 Expression of leading region genes in donors. Download FIG S3, PDF file, 0.2 MB. Copyright © 2021 Al Mamun et al. 2021 Al Mamun et al. https://creativecommons.org/licenses/by/4.0/ This content is distributed under the terms of the Creative Commons Attribution 4.0 International license.

### Deletion of *parA* or *parB* triggers the SOS response in donor cells.

**FIG 5:**
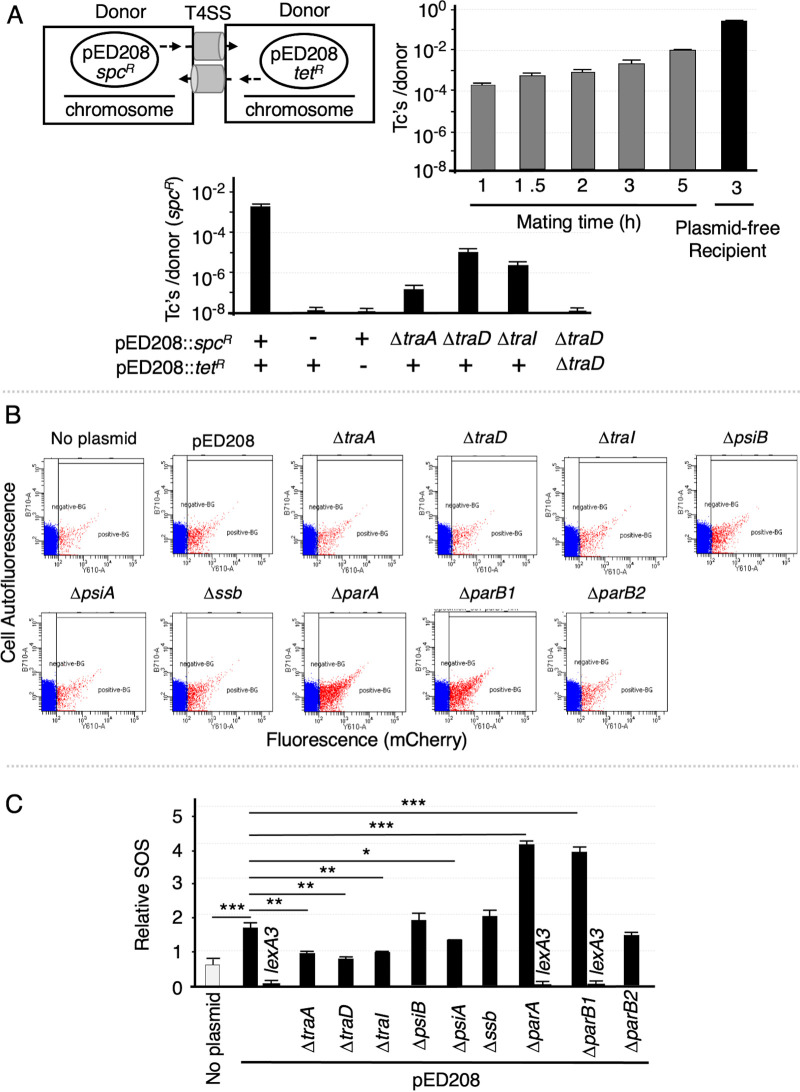
F plasmid carriage elicits the SOS response. (A) Schematic of self-mating assay showing matings between E. coli MC4100 strains carrying pED208::*spc*^r^ or pED208::*tet*^r^ mated for the times indicated and plated on media selective for Spc^r^ Tet^r^ transconjugants (Tc’s) or Tet^r^ (or Spc^r^) donors. (Bottom) Effects of *tra* gene deletions in one or both donor strains on self-mating frequencies in 3-h matings. Bottom rows indicate pED208 variants: (+), the pED208 variant shown at left; (-), donor lacking the respective plasmid, pED208 plasmids with the Δ*tra* mutations listed. Transfer frequencies are reported as the number of Spc^r^ Tet^r^ transconjugants per Spc^r^ donor (Tc’s/D). Results are reported as the mean frequency of transfer with standard error of mean (SEM). (B) Representative examples of flow cytometry data for the SOS reporter alone (No plasmid) or with the plasmids indicated. Panels depict flow cytometry data as described in the Fig. 3 legend. (C) Quantitation of the relative SOS responses elicited by strains presented in panel B (see Table S4 and data for the *lexA3* reporter strains in Fig. S2B). *P* values are shown above the bars as follows: *, *P* ≤ 0.05; **, *P* ≤0.005; ***, *P* ≤ 0.0005.

Finally, we asked whether simple carriage of an F plasmid stimulates SOS induction in a donor cell population. We envisioned this SOS response might be induced in plasmid-carrying populations as a result of redundant mating, which could generate transient ssDNA-inducing signals upon displacement of the T-strand from its complementary strand prior to exiting the cell or when a donor cell acquires the T-strand via homosexual mating (see reference 63). Despite safeguards against self-mating such as surface exclusion and incompatibility, F plasmids redundantly transfer among donor cells (64–66). Here, we found that redundant transfer of pED208 is in fact fairly robust. Upon mixing of donor strains harboring two fully functional pED208 plasmids differentially marked with *spc*^r^ or *tet*^r^, redundant transfer occurred at frequencies ranging from ∼10^−4^ to 10^−2^ Tcs/D in coincubations of 1 to 5 h (Fig. 5A). As expected, mutations blocking transfer of one plasmid diminished the frequencies of redundant transfer, while equivalent mutations in both plasmids yielded no transconjugants (Fig. 5A).

We introduced pED208 into the SOS reporter and assayed for SOS induction by flow cytometry. Interestingly, the pED208-carrying reporter exhibited an ∼1.7-fold-higher level of SOS induction than the plasmid-free reporter. An SOS response was not observed when the pED208-carrying reporter carried the *lexA3* mutation, indicative of true SOS induction (Fig. 5B and C and Fig. S2B). Reporters with pED208 deleted of *traD*, *traI*, or *traA* exhibited SOS responses commensurate with that of the parental SOS reporter, supporting the notion that redundant transfer among the donor cell population triggers the SOS response (Fig. 5B and C). We next asked whether the pED208-encoded maintenance functions under study modulated the SOS response in donor populations. Although we observed elevated SOS responses in strains harboring the Δ*psiB* or Δ*ssb* mutations relative to the pED208-carrying strain, the increases were not statistically significant (Fig. 5B and C). We suspect this is due to the overall lower frequency of plasmid transfer in donor-donor compared with donor-plasmid-free recipient matings. Interestingly, the Δ*parA* and Δ*parB* mutations conferred significantly elevated levels of SOS induction, which was not seen in parallel experiments using a *lexA3* reporter (Fig. 5B and C and Fig. S2B). It is unlikely, however, that the *par* mutations trigger SOS induction vis-á-vis homosexual transfer, since the Δ*parA* and Δ*parB* mutant plasmids failed to elicit SOS responses when delivered into plasmid-free recipients (Fig. 3). Instead, we propose that these mutations generate SOS-inducing signals in F plasmid-carrying cells through perturbing effects on plasmid replication and partitioning during cell division.

## DISCUSSION

Until now, the list of natural protein substrates of “dedicated” conjugation systems was restricted to relaxases (15, 34, 35), the SogL primase (67), the ParM partitioning protein (26), and FicT toxins of FicTA toxin-antitoxin modules (68). Except for R388-encoded TrwC, which exhibits relaxase and recombinase/integrase activities upon transfer to recipient cells (14), biological activities of translocated protein substrates have only been inferred. Here, we showed that several F-encoded “maintenance” proteins—ParA, ParB1, SSB, ParB2, PsiB, and PsiA—are translocated through the Tra_pED208_ T4SS and that translocated PsiB and SSB suppress the mating-induced SOS response. The SOS response entails the induction of proteins that promote the integrity of DNA, but it also includes error-prone factors that allow for improved survival of the cell but at the cost of elevated recombination and mutation (30, 31, 69). Conjugative elements such as F and other large conjugative plasmids that encode SOS inhibitor functions thus might have evolved the capacity to translocate such factors to preserve plasmid integrity and long-term survival in new transconjugants.

### Genetic requirements for conjugative protein translocation.

Our findings that the pED208 relaxosome must engage with TraD for translocation of all tested “maintenance” proteins confirm and extend previous findings from the Zechner lab. In studies of the R1-16 system, these investigators presented evidence that the R1-16 relaxosome-TraD interaction is a prerequisite for translocation of Cre-TraI and the mobilizable plasmids ColE1 and CloDF13, and for successful infection by R17 phage, which uses the R1-16 pilus to gain entry into the bacterial host (16, 43, 70).

Our findings suggest that pED208 relaxosome-TraD coupling is necessary for interbacterial transfer of all DNA and protein substrates, although not for phage infection as evidenced by M13 infection of relaxosome mutants. We acknowledge that the relaxosome-TraD interaction might have evolved as a signal to ensure the coordinated transfer of the F plasmid and the cohort of plasmid “maintenance” proteins under study here to avoid the energetically costly act of protein translocation in the absence of plasmid cotransfer. Whether F systems—or other conjugation systems—translocate proteins with other biological functions independently of relaxosome-TraD engagement remains to be determined.

Many large plasmids encode their own partitioning systems to ensure faithful transmission to both daughter cells during cell division (71). Intriguingly, in the Agrobacterium tumefaciens VirB/VirD4 system, the Par-like VirC1 and VirC2 proteins do not function in cell division but instead have been appropriated to stimulate transfer of the oncogenic T-DNA substrate to plant cells (51, 72). VirC1 and VirC2 act by forming a network of interactions with the VirD1 accessory factor and VirD2 relaxase, the T-DNA itself, and the VirD4 receptor, to spatially couple the T-DNA transfer intermediate with the polar-positioned VirB/VirD4 T4SS (51). Par-like factors are also required for transfer of chromosomal DNA through a Neisseria gonorrhoeae T4SS (52), and for conjugative transfer of plasmids R388 (53) and R1-16 (26). In the case of R1-16, *par* mutations also block attachment of bacteriophage R17 to otherwise active conjugative pili, creating phage resistance (26). Although we did not detect comparable requirements of ParA and ParB1 for pED208 transfer or M13 phage infection, our findings are consistent with a model in which ParA and ParB1 function similarly to Par proteins such as VirC1 and VirC2 in physically coupling secretion substrates with the TraD receptor. To reconcile the observed stimulatory effects of the Par proteins on protein but not pED208 plasmid transfer, we note that pED208’s *tra* operon is abundantly expressed due to an IS element insertion, which results in hyperpiliation and high-frequency plasmid transfer (23). We suspect that Tra_pED208_ T4SS overproduction masks the stimulatory effects of the Par proteins on DNA trafficking, but due to the comparative inefficiency of CRAfT, we were still able to detect Par stimulation of protein translocation.

Interestingly, coupling of the relaxosome with VirD4 receptors is not a general requirement for translocation of protein substrates through other conjugation systems. For example, the pKM101 plasmid-encoded Tra system engineered with a chimeric VirD4 receptor transfers nonnative protein substrates to other bacteria in the absence of plasmid cotransfer (19). Similarly, the IncI1 plasmid Col1b-P9 transfers the SogL primase in several hundred copies to target cells independently of the plasmid (67). In this context, it is striking that systems closely related to the pKM101 and Col1b-P9 transfer systems have been extensively appropriated over evolutionary time for deployment as effector translocators, the two best-characterized being the A. tumefaciens VirB/VirD4 and L. pneumophila Dot/Icm systems (1, 33, 73, 74). In contrast, no effector translocators have yet been identified with signatures of F-encoded conjugation machines. It is enticing to propose that nature has selectively adapted ancestral pKM101(VirB/VirD4)- and Col1b-P9(Dot/Icm)-like machines—and not F systems—for deployment as effector translocators at least in part because of their relaxed machine activation requirements.

### Conjugative protein translocation suppresses the mating-induced SOS response.

E. coli Hfr strains carry F plasmids integrated in their chromosomes, and matings involving chromosomal transfer to E. coli and *S.* Typhimurium recipients confirmed that conjugation induces the SOS regulon (30). This response was stronger in interspecies matings, but the medical importance of the mating-induced SOS response in intraspecies matings is underscored by evidence for SOS-induced emergence of antibiotic resistance development as a result of mutations introduced through error-prone DNA replication (75) as well as SOS-directed transcriptional activation of integrases and shuffling of integron cassettes (31). By use of the P*_sulA_*-*mCherry* reporter and single-cell imaging, we confirmed that pED208 transfer induces the SOS response in recipient cells, and we also showed that transfer of pED208Δ*psiB* confers a significantly elevated response. Importantly, transfer of pED208Δ*ssb* also elicits a strong SOS response, establishing for the first time that plasmid-encoded SSB also plays a role in blocking SOS induction in new transconjugants. Although pED208-carrying donors activated the SOS response at levels above the “red” gate in a small fraction (∼0.2%) of the total recipient cell population, we note that single-cell imaging almost certainly underestimates the fraction of new transconjugants exhibiting some level of SOS induction. This is supported by the fact that flow cytometry captures only a temporal snapshot of the SOS response during mating and by results of single-cell imaging analyses showing that (i) cells exhibit a highly variable SOS response when exposed to DNA damaging agents (57) and (ii) only a small fraction of cells harboring the SOS-constitutive *recG* mutation display a detectable SOS response (Fig. 3B and C and Table S3) (57, 58, 61). Moreover, in our studies, SOS-induced fluorescence was quantitated as a fraction of the total recipient cell population, yet donors transfer pED208 to only 1 in 10 to 100 recipient cells in 1-h matings. Assuming that most or all P*_sulA_*-activated cells correspond to new transconjugants, in fact an appreciable fraction (∼2 to 20%) of new transconjugants are predicted to exhibit a detectable SOS response.

Transfer of the Δ*psiB* or Δ*ssb* mutant plasmids triggered a strong SOS response, despite the fact that these plasmids, respectively, carry wild-type *ssb* or *psiB* genes. Why, then, does production of PsiB by the Δ*ssb* mutant or SSB by the Δ*psiB* mutant not suppress the SOS response? A trivial explanation is that the Δ*psiB* and Δ*ssb* mutations have polar effects on expression of other genes in the *ssb-parB2-psiB-psiA* cluster. We think this is unlikely because *trans-*expression of *psiB* or *ssb* in the respective pED208Δ*psiB* or pED208Δ*ssb* donor strains strongly suppressed the mating-induced SOS response. Instead, we propose that PsiB and SSB act synergistically to suppress the mating-induced SOS response in recipient cells. This model is in line with results of an early study implicating functional interactions between PsiB and SSB in suppressing high SOS levels conferred by *recA441* and *recA730* mutations (76). This functional interaction is complex, because on the one hand, SSB binding to the incoming T-strand can block RecA from binding this ssDNA substrate independently of PsiB (77). On the other hand, once SSB binds an ssDNA substrate, it can melt ssDNA secondary structure and stimulate RecA-ssDNA nucleation (78); however, this in turn stimulates PsiB function because PsiB directly binds RecA and specifically prevents it from binding SSB-coated ssDNA (79, 80). The concerted actions of PsiB and SSB might mount a more effective block against formation of SOS-inducing RecA-ssDNA filaments than achieved with either translocated protein alone.

For PsiB and SSB to suppress the SOS response in new transconjugants, translocation of both substrates must occur within a kinetic window and at levels sufficient to block RecA−T-strand filamentation. Available RecA is abundantly present in cells (56), implying that many hundreds of copies of both SSB and RecA-binding PsiB must be translocated. The number of molecules of translocated PsiB need not match available RecA, however, because PsiB effectively inhibits RecA-driven SOS induction even when it is present at comparatively low concentrations (79). These findings led the authors to propose that a threshold in the number of RecA-ssDNA filaments is required for SOS induction, and PsiB exerts its effects by blocking formation of this critical threshold (79). It is known that T4SSs are capable of translocating hundreds of different effectors, e.g., L. pneumophila Dot/Icm system (81), as well as effectors in hundreds to thousands of copies, e.g., Col1b-P9-encoded SogL primase (67). Perhaps most relevant to our present findings, the A. tumefaciens VirB/VirD4 T4SS is estimated to deliver the VirE2 effector (an SSB) in thousands of copies to plant cells. Upon translocation, the VirE2 SSB cooperatively binds and protects the single-stranded T-DNA intermediate, which is ∼15 to 30 kb in length but can exceed 200 kb, from degradation during transit to the plant nucleus (82–84). We confirmed that pED208-encoded SSB is produced from its native promoter at detectable levels in donor cells but acknowledge that further quantitative comparisons of natively synthesized PsiB and SSB in donors and new transconjugants are needed to assess the biological impacts of protein translocation. Nevertheless, at this juncture, there is sufficient precedent supporting our proposal that Tra_pED208_ T4SS coordinates the trafficking of these SOS inhibitors kinetically rapidly and at levels sufficient to block the mating-induced SOS response.

Importantly, the translocated forms of PsiB and SSB need not be entirely responsible for suppression of the SOS response in new transconjugants. In work initiated in the 1990s, evidence was presented for the existence of imperfect, inverted repeat sequences upstream of the *ssb*-*parB2-psiB-psiA* clusters of F and ColIb-P9 model plasmids. Single-stranded forms of these regions were shown to adopt stem-loop structures to which RNA polymerase binds and synthesizes downstream transcripts (85–87). Transcripts generated from these single-stranded promoters, designated F*rpo* or *ssi* (single-strand initiation), can be translated, or their 3′ ends can serve as priming sites for DNA synthesis (85). These *in vitro* findings led to a proposal that, upon transfer of the T-strand, F*rpo* promoters form and subsequent translation yields PsiB at the levels necessary for SOS inhibition. Indeed, transient transcription of *psiB* was demonstrated in new transconjugant cells (88, 89), although neither the kinetics of protein synthesis versus DNA transfer nor the amount of PsiB produced from F*rpo-*directed gene expression was examined. Deciphering the relative contributions of the T4SS-directed protein delivery versus F*rpo*-mediated gene expression pathways to SOS suppression requires further study, but we suggest the two pathways might in fact be spatiotemporally integrated. In an appealing two-stage model, (i) immediately upon establishment of the productive “mating junction,” PsiB and SSB are translocated through the T4SS to recipient cells where they initiate SOS suppression by binding available RecA and the incoming T-strand, and then (ii) upon formation of F*rpo* and recruitment of RNA polymerase, transcription of the leading region genes generates additional copies of the SOS inhibitors. In the context of this model, it is notable that RNA polymerase initiates transcription from F*rpo* promoters specifically when the ssDNA is coated with SSB (85). The translocated form of SSB thus might function dually, by coordinating with translocated PsiB to initiate SOS suppression and by binding F*rpo* to stimulate recruitment of RNA polymerase for an amplified SOS-suppressive response. In line with this two-stage model, we observed that SSB-Str accumulated at slightly higher levels in donor-recipient mating mixes than in the donor-only cell population, possibly reflecting the sum of protein synthesis in donors and new transconjugants.

In summary, results of our studies expand the repertoire of known proteins and associated biological functions that are translocated through “dedicated” conjugation systems. Our findings have potential therapeutic applications, insofar as deployment of conjugation systems for translocation of SOS inhibitors such as PsiB and SSB in infection settings might pose an effective block against the emergence of SOS-induced recombination or mutagenesis as drivers of antibiotic resistance.

## MATERIALS AND METHODS

### Bacterial strains, primers, plasmids, and media.

E. coli strains were grown in Luria-Bertani (LB) medium at 30°C for recombineering and 37°C for other applications. Carbenicillin, kanamycin, tetracycline, chloramphenicol, and rifampicin (Sigma) were used at final concentrations of 100, 50, 20, 20, and 100 μg/ml, respectively. Oligonucleotides (Sigma) used for sequence amplifications are listed in Table S2 in the supplemental material. Restriction endonucleases, T4 DNA ligase, phusion DNA polymerase, *Taq* DNA polymerase, and deoxynucleoside triphosphates (dNTPs) were from New England Biolabs Inc. DNA polymerase red mix was from Genesee. pED208 genes and the *oriT* sequence were deleted, and streptactin (Strep)-tagged *ssb* was introduced, by substitution with kanamycin resistance (*kan*^r^) gene cassette using standard recombineering procedures (90). The *kan*^r^ gene was excised using the temperature-sensitive plasmid pCP20 that expresses yeast Flp recombinase (91). To substitute C-terminally Strep-tagged *ssb* for wild-type *ssb* on pED208, Strep-kanamycin (kan) was amplified using the primers ssbStrep_F and ssbStrep_R using pKD13 as a template, and inserted in the pED208 by short homology. The insertion was verified by using the primers that were used to check for the Δ*ssb* mutation. Gene disruptions were confirmed by PCR amplification followed by sequencing across the deletion junctions. Nonpolarity of the gene mutations was confirmed by complementation with the corresponding gene expressed from the P*_BAD_* promoter.

10.1128/mBio.01629-21.2 TABLE S2 Description of primers. Download Table S2, PDF file, 0.2 MB. Copyright © 2021 Al Mamun et al. 2021 Al Mamun et al. https://creativecommons.org/licenses/by/4.0/ This content is distributed under the terms of the Creative Commons Attribution 4.0 International license.

### Plasmid constructions.

pED208 genes of interest were expressed from the P*_BAD_* promoter by amplification of the respective genes with oligonucleotides listed in Table S2 and pED208 as the template. PCR products were digested with NheI and HindIII, and the resulting fragments were inserted into similarly digested pBAD24. Plasmids expressing *cre* fused to pED208 genes were constructed as follows. Plasmid pAM38 expressing *cre* from the P*_BAD_* promoter was constructed by amplification of *cre* from pTB33 (Table S1), the PCR product was amplified to carry an NheI site at the 5′ end and XbaI and HindIII sites at the 3′ end, and the resulting fragment was inserted into NheI/HindIII-digested pBAD24. pED208 genes were amplified with 5′ and 3′ primers carrying XbaI and HindIII sites, the PCR products were digested with XbaI and HindIII, and the resulting fragments were inserted into similarly digested pAM38. Plasmid constructs were confirmed by sequencing across the entire *tra* genes or *cre-tra* fusions. A plasmid carrying pED208’s origin-of-transfer (*oriT*) region (25), designated pAM118 or p*oriT*_pED208_, was generated by PCR amplification of the *oriT* sequence, digestion of the PCR product with KpnI and HindIII, and insertion of the resulting fragment into similarly digested pBAD24. Similarly, full-length and C-terminal 15-residues-deleted *traD* gene were cloned into the KpnI/HindII sites of pBAD24, and named pAM110 and pAM112, respectively.

10.1128/mBio.01629-21.1 TABLE S1 Strains and plasmids used in this study. Download Table S1, PDF file, 0.2 MB. Copyright © 2021 Al Mamun et al. 2021 Al Mamun et al. https://creativecommons.org/licenses/by/4.0/ This content is distributed under the terms of the Creative Commons Attribution 4.0 International license.

### RT-PCR.

Real-time reverse transcription-PCR (RT-PCR) was carried out to analyze expression of *psiB*, *psiA*, and *ssb* from pED208 in donor cells. For isolation of total RNA, an overnight culture of E. coli MC4100(pED208) was diluted 1:50 in fresh LB medium and incubated with shaking for 1.5 h, then growth was stopped by placing cultures on ice. RNA from 1.5-ml culture was isolated with the Direct-zol RNA Miniprep kit (catalog no. R2050T; Zymo Research) as described by the vendor. After elution, DNase I (NEB) was added to the samples and incubated at 37°C for 1 h, and RNA was purified with Zymo Research minicolumns according to the manufacturer’s protocol using RNase-free water for elution. RNA integrity was confirmed by the presence of clearly defined rRNA bands on agarose gels and later quantified for concentration by optical density at 260 nm (OD_260_) readings. DNA contamination was further assessed by amplifying ∼100-bp fragment of the *gyrA* gene. Very little or no PCR products for RNA samples compared with that of a DNA template was used for RT-PCR. For RT-PCR, primers were designed using the IDT (Integrated DNA Technologies) Primer Quest Tool. The primer sequences are listed in Table S2. Three hundred nanograms of total RNA was reverse transcribed using iScript cDNA Synthesis kit (catalog no. 1708891; Bio-Rad) following the manufacturer’s protocol. The reaction mixtures for RT-PCR were prepared using iTaq Universal SYBR Green Supermix (catalog no. 172-5121; Bio-Rad) according to the vendor. For amplification, a CFX06TM real-time PCR system (Bio-Rad) was used with the following PCR program (1 cycle, 95°C for 10 min; 40 cycles, with 1 cycle consisting of 95°C for 15 s, 60°C for 30 s, and 72°C for 15 s). Changes in gene transcription were calculated using the comparative threshold cycle (*C_T_*) (2^−ΔΔCt^) method (92). Results were normalized to the housekeeping reference gene 16S rRNA.

### Cre recombinase assay for translocation (CRAfT).

The Cre reporter was used to assay for translocation of pED208-encoded proteins through the Tra_pED208_ T4SS into strain CSH26Cm::LTL, which contains a *loxP-tet*^r^-*loxP* cassette interrupting a *chl*^r^ gene on the bacterial chromosome (70). Cre-mediated excision of the *loxP* cassette restores integrity of the *chl*^r^ gene in recipient cells, conferring a Chl^r^ Tet^s^ phenotype. For the Cre reporter assay for translocation (CRAfT), strains were grown overnight in LB broth with antibiotic selection at 37°C with shaking. Overnight cultures were inoculated 1:50 into fresh LB broth (2 ml), and the culture was grown at 37°C with shaking for 1.5 h (OD_600_ of ∼0.3). Donor and recipient cells (10 μl) each were mixed, spotted onto sterile nitrocellulose filters on LB plates containing 0.2% arabinose (final concentration) for induction of *cre* gene fusions from the P*_BAD_* promoter, and incubated at 37°C for the times indicated. Cells were resuspended from the filter in LB broth and serially diluted, and donors, recipients, and *loxP* recombinants were selected on LB agar plates containing the appropriate antibiotics. The frequency of Cre recombination was calculated as the number of recombinants per donor (Rcs/D). Experiments were performed at least three times in triplicate, and results are reported as the mean frequency of transfer with standard error of the mean (SEM).

### Conjugation assays.

Donor and recipient cells were grown overnight at 37°C in the presence of the appropriate antibiotics, diluted 1:50 in fresh antibiotic-free LB medium, and incubated without shaking for 1.5 h. Donor and recipient cell cultures (75 μl each) were mixed and incubated without shaking for 1.5 h, and if necessary, cells were induced with arabinose (0.2% final concentration). For time course experiments, mating mixtures were placed on ice and vortexed for 30 s to disrupt mating at the indicated time of incubation. Mating mixtures were serially diluted and plated onto LB agar containing antibiotics selective for transconjugants (Tcs) and donors. The frequency of DNA transfer was calculated by dividing the number of Tc colonies with the number of donor colonies (Tcs/D). For donor-donor matings, E. coli strains harboring pED208::*spc*^r^ or pED208::*tet*^r^ were mated in broth for the times indicated and mating frequencies were reported as Tcs per Spc^r^ or Tet^r^ donor. Mating experiments were performed at least three times in triplicate, and results are reported as the mean frequency of transfer with standard error of the mean (SEM). To assess SSB protein production in donor cells or mating mixes, E. coli MC4100(pED208::*ssb-str*), MC4100(pED208), and MC4100Cm were cultivated as described above. Cultures of pED208-carrying donor strains were spotted (10 ml) alone or together with MC4100Cm recipients (10 ml) on nitrocellulose filter discs on LB agar plates and incubated for 3 h. Filters were resuspended in 1 ml of LB and vortexed, and suspended samples were mixed with 2× Laemmli’s sample buffer and analyzed by immunostaining for SSB-Str production.

### Western blotting.

Production of SSB-Str by pED208::*ssb-str* donors only or donor-recipient mating mixes was assessed by Western blot analyses. Briefly, cell lysates were loaded on a sodium dodecyl sulfate (SDS)-polyacrylamide gel on a per cell equivalent basis prior to electrophoresis, Western transfer, and immunostaining with antistreptactin (anti-Strep) antibodies. As a loading control, blots were developed with antibodies against the β subunit of E. coli RNA polymerase. Quantifications of the immunostained bands were performed by densitometry using Image J software, with levels in the SSB-Str-producing donor strain set at 1.

### Assays for F pilus production.

F pilus production was assessed by assaying for the presence of TraA pilin in material sheared from the cell surface by immunoblot analyses. For total cell protein, 5-ml cell cultures were grown overnight in LB medium with appropriate antibiotic selections, and cell pellets from 1-ml culture volumes were resuspended in 100 μl of Laemmli’s sample buffer and boiled for 5 min. F pili were recovered from the culture supernatants by polyethylene glycol 8000 (PEG 8000) precipitation as previously described (93). Precipitated pili were harvested by centrifugation at 15,000 × *g* for 30 min, resuspended in 100 μl Laemmli’s sample buffer, and boiled for 5 min. Total cellular protein and material in the F pilus preparations were electrophoresed through SDS−15% polyacrylamide (30:0.8 acrylamide/bis-acrylamide) gels, and transferred to nitrocellulose membranes. TraA pilin was detected by development of immunoblots with anti-TraA antibodies specific for pED208 TraA (kindly provided by L. Frost) and horseradish peroxidase (HRP)-conjugated secondary antibodies followed by chemiluminescence. As a loading control, blots were also developed with antibodies to the β subunit of E. coli RNA polymerase (BioLegend). F pilus production was further confirmed by infection with bacteriophage M13, which uses the F pilus as a receptor. For phage infection, 30 μl of the cells grown overnight were plated on LB-agar plates (supplemented with antibiotics, and 0.2% arabinose where necessary). After the plates were dried, they were spotted with 2 μl of M13 (titer of 10^11^ phage/ml) and incubated overnight at 37°C. Plaque formation served as an indicator of F pilus production (94). Finally, F pilus-mediated aggregation was determined as previously described (24). A cohesion index reflecting the extent of cell aggregation was calculated as described previously (24), and results were reported as “+” for no significant difference or “-” for significantly different from MC4100(pED208), as determined by Student’s *t* tests.

### Detection of the mating-induced SOS response by flow cytometry.

Induction of the SOS response during mating was quantitated at the single-cell level by flow cytometry as previously described (57). E. coli MG1655 expressing red fluorescent protein mCherry from the SOS-inducible *sulA* promoter (58) (Δ*att*λ::P*_sulA_*-*mCherry*) served as a recipient during mating. Matings between MC4100 strains carrying pED208 or mutant plasmids and the SOS reporter strain were carried out as described above, and mating mixtures were harvested by centrifugation and resuspended in 1 ml of filter-sterilized M9 minimal salts medium. Mating mixtures were diluted 1:25 to 1:50 with M9 minimal salts, samples were subjected to flow cytometry using an LSR Fortessa flow cytometer (BD Biosciences), and data were analyzed with BD FACSDiva and FlowJo software. A “red” gate was set using the control *lexA3* mutant (SOS induction-deficient) cells; cells to the right of the gate are considered SOS induced (57, 58). For these analyses, 10^6^ events were collected per strain, with each strain assayed three times in three independent experiments. The percentage of SOS-induced cells was calculated by dividing the number of SOS-induced cells with 10^6^ events multiplied by 100 [(number of SOS-induced cells/10^6^) × 100] (Tables S3 and S4). The relative SOS induction was determined by dividing the percentage of SOS induction for the F^+^ × SOS reporter mating by the percentage of SOS induction of the control mix (F^−^ donor × SOS reporter). For redundant plasmid transfer, the relative induction of SOS was determined by dividing the percentage of SOS induction of the F^+^ SOS reporter by that of the F^−^ SOS reporter. Experiments were replicated three times in duplicate, and results for a representative experiment are presented.

### Statistical analysis.

Experiments were performed at least three times in triplicate, and results are reported as the mean frequency of transfer with standard error of the mean (SEM). Unless indicated otherwise, *P* values were determined by two-tailed Student’s *t* test for strains harboring pED208 mutant variants compared with that the isogenic strain carrying WT pED208 in the same experiments.

### Data availability.

We declare that all other data supporting the findings of this study are available within the paper and its supplemental material files.
