# Supplementary material for: Protein Transfer through an F Plasmid-Encoded Type IV Secretion System Suppresses the Mating-Induced SOS Response
Source: mBio. 2021 Jul 13;12(4):e01629-21. doi: 10.1128/mBio.01629-21 (PMC8406263; doi:10.1128/mBio.01629-21)
Supplement: FIG S1 [file mbio.01629-21-sf001.pdf]

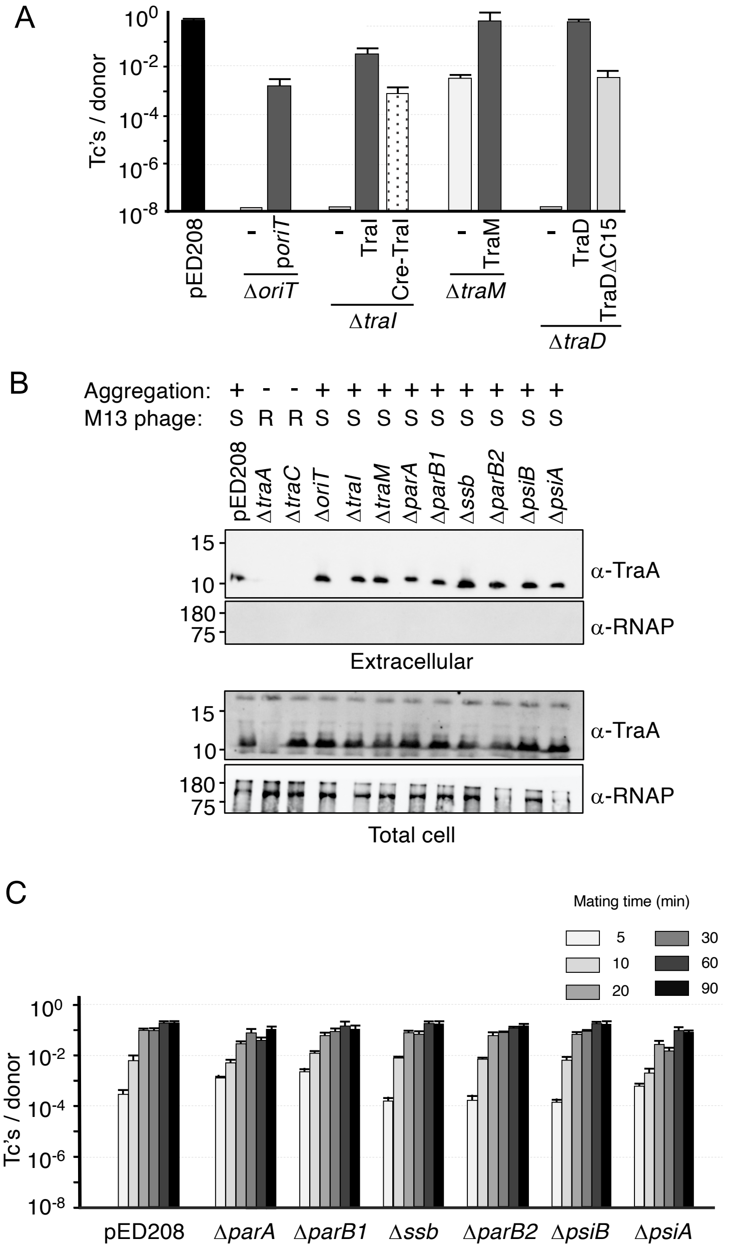

Fig. S1. Phenotypes of pED208 mutations. **A)** Effects of deletion mutations shown on pED208 transfer. WT, pED208. Donors harboring pED208Δ*oriT* transfer a p*oriT*$_{\mathrm{pED208}}$ plasmid. Complementation of the Δ*traI*, Δ*traM,* and Δ*traD* mutations by *trans*-expression of the corresponding genes restores transfer of pED208 mutant plasmid. Cre-TraI production supports transfer of the Δ*traI* mutant plasmid and TraDΔC15 production partially supports transfer of the Δ*traD* mutant plasmid. **B)** Assays for production of the F pilus. Top: F pilus-mediated aggregation as monitored with a cohesion assay (see Materials and Methods. +, Pilus-mediated aggregation; -, no aggregation. Susceptibility to infection by bacteriophage M13, which uses the F pilus as a receptor. S, sensitive; R, resistant. Lower: Presence of TraA pilin in the supernatant of sheared cells. The total cell lysate or material recovered in the supernatant was subjected to SDS-PAGE, and immunoblots were stained with antibodies specific for pED208-encoded TraA pilin (α-TraA) or cytoplasmic RNA polymerase (α-RNAP) as a control for cell lysis. Left: Molecular sizes (in kDa) of protein markers. **C)** Effects of the deletion mutations shown on pED208 transfer frequencies in liquid matings ranging from 5 to 90 minutes. Matings were disrupted by vortexing, then placing the mating mixtures on ice prior to plating on media selective for growth of transconjugants (Tc's) or Donors. Panels A and C: Tc's/D, Transconjugants/Donor. Results are reported as the mean frequency of transfer with standard error of mean (SEM) shown.
