# Supplementary material for: Protein Transfer through an F Plasmid-Encoded Type IV Secretion System Suppresses the Mating-Induced SOS Response
Source: mBio. 2021 Jul 13;12(4):e01629-21. doi: 10.1128/mBio.01629-21 (PMC8406263; doi:10.1128/mBio.01629-21)
Supplement: TABLE S3 [file mbio.01629-21-st003.pdf]

Table S3. SOS induction during mating[a]

MC4100 Donor	MG1655 Recipient: SOS Reporter	% of SOS-activated cells (Av±SEM)
No plasmid	WT	0.09±0.01
No plasmid	*lexA3*	0.01±0.01
pED208	WT	0.18±0.02
pED208	*lexA3*	0.003±0.001
pED208Δ*ssb*	WT	0.6±0.05
pED208Δ*ssb*	*lexA3*	0.005±0.001
pED208Δ*parB2*	WT	0.18±0.02
pED208Δ*parB2*	*lexA3*	0.007±0.001
pED208Δ*psiB*	WT	0.62±0.14
pED208Δ*psiB*	*lexA3*	0.01±0.002
pED208Δ*psiA*	WT	0.13±0.02
pED208Δ*psiA*	*lexA3*	0.004±0.001
pED208Δ*parA*	WT	0.16±0.013
pED208Δ*parA*	*lexA3*	0.006±0.0002
pED208Δ*parB1*	WT	0.17±0.01
pED208Δ*parB1*	*lexA3*	0.004±0.0002
pED208Δ*traD*	WT	0.08±0.002
No Donor	*recG*	1.2±0.14

[a] % of SOS expressing cells = [# of SOS-activated cells/# of cells sorted (10^6)] x 100 x 2. We multiplied by 2 to normalize for mixing of equal number of donor cells with the SOS reporter recipient cells for comparisons of SOS expression by donor cells only shown in Table S4. Av, average of three independent experiments; each experiment performed in triplicate. SEM, standard error of mean.
