# Supplementary material for: Protein Transfer through an F Plasmid-Encoded Type IV Secretion System Suppresses the Mating-Induced SOS Response
Source: mBio. 2021 Jul 13;12(4):e01629-21. doi: 10.1128/mBio.01629-21 (PMC8406263; doi:10.1128/mBio.01629-21)
Supplement: FIG S2 [file mbio.01629-21-sf002.pdf]

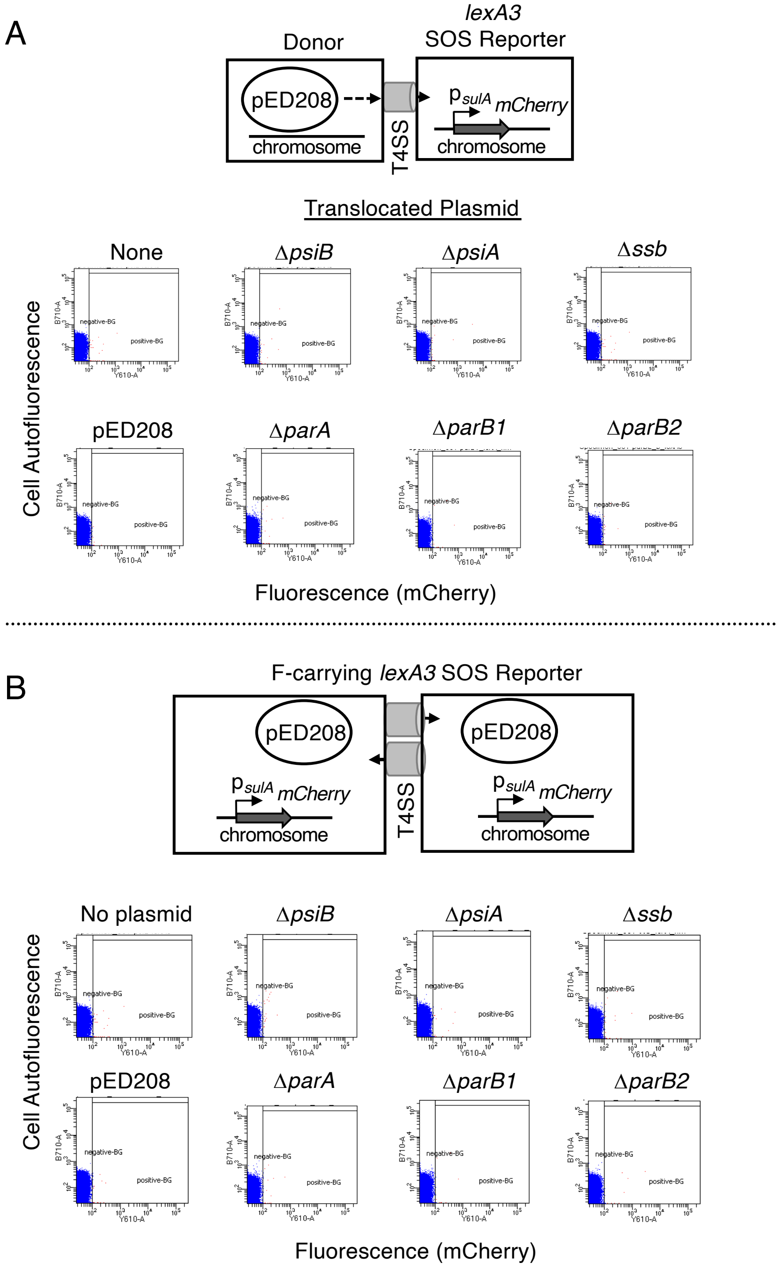


**Fig. S2.** SOS responses elicited by pED208 variants in the *lexA3* SOS reporter strain. **A)** Schematic showing the MC4100(pED208) donor mated with MC4100 harboring the *lexA3* SOS reporter. Lower: Representative examples of flow cytometry data, reflecting transcriptional induction of the SOS-inducible $P_{sulA}$ promoter. For data presented in Fig. 3, the 'red' gates (SOS-uninduced) demarking flow-cytometry events (cells) colored red to the right (SOS induced) and blue to the left (SOS-uninduced) were set using the SOS-uninducible *lexA3* mutant strain shown. Panels depict flow cytometry data for donors harboring, no plasmid, WT pED208, or the pED208 variants with gene deletions listed. **B)** Schematic showing the *lexA3* SOS reporter lacking or carrying WT pED208. Lower: Representative examples of flow cytometry data*,* reflecting transcriptional induction of the SOS-inducible $P_{sulA}$ promoter. For data presented in Fig. 5, the 'red' gates (SOS-uninduced) demarking flow-cytometry events (cells) colored red to the right (SOS induced) and blue to the left (SOS-uninduced) were set using the SOS-uninducible *lexA3* mutant strain shown. Panels depict flow cytometry data for strains harboring no plasmid, WT pED208, or the pED208 variants with gene deletions listed.
