# Supplementary material for: Protein Transfer through an F Plasmid-Encoded Type IV Secretion System Suppresses the Mating-Induced SOS Response
Source: mBio. 2021 Jul 13;12(4):e01629-21. doi: 10.1128/mBio.01629-21 (PMC8406263; doi:10.1128/mBio.01629-21)
Supplement: TABLE S4 [file mbio.01629-21-st004.pdf]

Table S4. SOS activation in plasmid-carrying populations[a]

MG1655 Reporter Strain	% of SOS-activated cells (Av±SEM)[a]
No plasmid	0.07 ± 0.003
pED208	0.11 ± 0.01
pED208 (*lexA3* Reporter Strain)	0.003 ± 0.0004
pED208 Δ*ssb*	0.15 ± 0.02
pED208 Δ*parB2*	0.12 ± 0.01
pED208 Δ*psiB*	0.14 ± 0.02
pED208 Δ*psiA*	0.1 ± 0.01
pED208 Δ*parA*	0.3 ± 0.01
pED208 Δ*parA* (*lexA3*Reporter Strain)	0.003 ± 0.001
pED208Δ*parB1*	0.29 ± 0.02
pED208Δ*parB1* (*lexA3* Reporter Strain)	0.003 ± 0.001
pED208Δ*traA*	0.07 ± 0.005
pED208Δ*traD*	0.06 ± 0.003
pED208Δ*traI*	0.07 ± 0.002

[a] % of SOS-activated cells = [# of SOS-activated cells/# of cells sorted (10^6)] x100. Av, average of three independent experiments, each experiment performed in triplicate. SEM, standard error of mean.
