# Supplementary material for: Protein Transfer through an F Plasmid-Encoded Type IV Secretion System Suppresses the Mating-Induced SOS Response
Source: mBio. 2021 Jul 13;12(4):e01629-21. doi: 10.1128/mBio.01629-21 (PMC8406263; doi:10.1128/mBio.01629-21)
Supplement: FIG S3 [file mbio.01629-21-sf003.pdf]

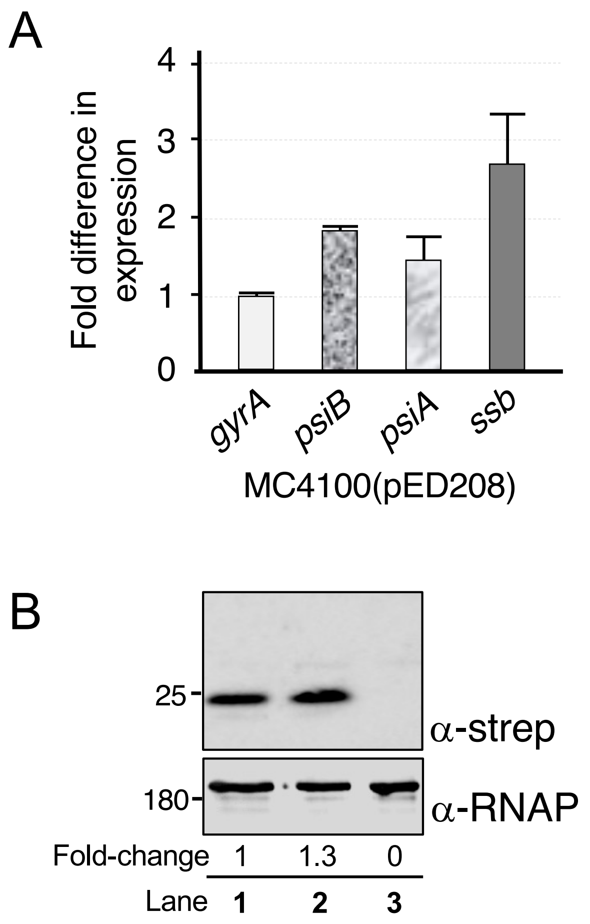

1 pED208::*ssb-str* donors only

2 pED208::*ssb-str* donors + recipients (mating mix)

3 pED208 donors + recipients (mating mix)

Fig. S3. Expression of 'maintenance' genes in MC4100(pED208) cells. **A)** Expression of *psiB, psiA* and *ssb* genes as measured by real-time RT-PCR. Template RNA was isolated from MC4100(pED208). *psiB, psiA*, and *ssb* transcript levels were normalized to that of 16S rRNA, and reported relative to *gyrA* transcript levels. Experiments were repeated 3 times, and the average values are presented. SE, standard error of mean. **B)** Steady-state abundance of strep-tagged SSB (SSB-Str) in donors harboring pED208::*ssb-str* (lane 1), a mating mix composed of MC4100(pED208::ssb-str) donors and MC4100Cm recipients (lane 2), and a mating mix composed of MC4100(pED208) and MC4100Cm recipients (lane 3). Fold-change of SSB-Str levels in the mating mix relative to the pED208::*ssb-str* donors was determined by densitometry as described in the Materials and Methods.
