# Supplementary material for: Protein Transfer through an F Plasmid-Encoded Type IV Secretion System Suppresses the Mating-Induced SOS Response
Source: mBio. 2021 Jul 13;12(4):e01629-21. doi: 10.1128/mBio.01629-21 (PMC8406263; doi:10.1128/mBio.01629-21)
Supplement: TABLE S2 [file mbio.01629-21-st002.pdf]

Table S2. Description of primers

Primer	Sequence (5' to 3')[a]	Purpose
Del-*traI*-FRT-Kan_F	cagggcagactgcctgatggcagtgacctgtcccggatggttga tggtgtcATTCCGGGGATCCGTCGACC	pED208::*spc*$^R\Delta$*traI*
Del-*traI*-FRT-Kan_R	ccggcaatgccggcagcagatcctgccgggcctgctccgccag ctcctgggcTGTAGGCTGGAGCTGCTTCG	pED208::*spc*$^R\Delta$*traI*
Del-*psiB*-FRT-Kan_F	cggaggccgccccttcgggggcggcagtaaggagaacatgat gcgtaacATTCCGGGGATCCGTCGACC	pED208::*spc*$^R\Delta$*psiB*
Del- *psiB*-FRT-Kan_R	tgataacctccggcgaccaggctggcagcgtgcagatgacaga gaaaggTGTAGGCTGGAGCTGCTTCG	pED208::*spc*$^R\Delta$*psiB*
Del- *psiA*-FRT-Kan_F	acctgtcactggtccctctgagtcatgagcgtcgtgcggtaatgc aggccATTCCGGGGATCCGTCGACC	pED208: *spc*$^R\Delta$*psiA*
Del- *psiA*-FRT-Kan_R	atcatccagcgttccatcgatgccagtgacggaaaacgctcactc cagcgTGTAGGCTGGAGCTGCTTCG	pED208::*spc*$^R\Delta$*psiA*
Del-*ssb*-FRT-Kan_F	gtccgttata tgcccaatgg cggcgcggtc gctaatctca gcctggccac ATTCCGGGGATCCGTCGACC	pED208: *spc*$^R\Delta$*ssb*
Del-*ssb*-FRT-Kan_R	ctgacggggt tttctgccgc ccttcgcttt cggggatttt tcaggagtgc TGTAGGCTGGAGCTGCTTCG	pED208::*spc*$^R\Delta$*ssb*
Del-*parA*-FRT-*cat*_F	ttaaggactg ttacatgaca ctgaaaatag gacctgtcgg caaacttatc GTGTAGGCTG GAGCTGCTTC	pED208: *spc*$^R\Delta$*parA*
Del- *parA*-FRT-*cat*_R	tccctgaagg atctcgatga ggagattcag attcagtgta tcaataggcc CCT CCTTAGTTCC TATTCCGAAG TTCC	pED208::*spc*$^R\Delta$*parA*
Del- *parB1*-FRT-*cat*_F	gacgactc caggcgcagc aggaaatgaa aaaaaaagca tctgttgttt GTGTAGGCTG GAGCTGCTTC	pED208: *spc*$^R\Delta$*parB1*
Del- *parB1*-FRT-*cat*_R	ctgcccaaag gcttggttgg ttttttgttc tgagccttag atttcgcatg CCT CCTTAGTTCC TATTCCGAAG TTCC	pED208::*spc*$^R\Delta$*parB1*
Del- *parB2*-FRT-Kan_F	cca aagcgtcaac gcataaagct gatgctcagt cagacgtcct ggttgcc ATTCCGGGGATCCGTCGACC	pED208: *spc*$^R\Delta$*parB2*
Del- *parB2*-FRT-Kan_R	aacagcacgt tcaacaccat caagatcact gcgtgtggta tgtcccatcg TGTAGGCTGGAGCTGCTTCG	pED208::*spc*$^R\Delta$*parB2*
Del-*traM*-FRT-Kan_F	gacaaatttaaatttctaataaggtttttgaaatgccaaaaatacag ATTCCGGGGATCCGTCGACC	pED208::*spc*$^R\Delta$*traM*
Del-*traM*-FRT-Kan_R	cggaatcagggaaatgattccggcttttagaaatgaaaccaggtt cactcTGTAGGCTGGAGCTGCTTCG	pED208::*spc*$^R\Delta$*traM*
Del-*oriT*-FRT-Kan_F	ttagccagagtgccatttttcataaaaaacggcaatgcccggaaa tactATTCCGGGGATCCGTCGACC	pED208::*spc*$^R\Delta$*oriT*
Del-*oriT*-FRT-Kan_R	tagtcacatgtgaatcggccatttattttattagagtcagtctggatt caTGTAGGCTGGAGCTGCTTCG	pED208::*spc*$^R\Delta$*oriT*
Del-*traD*-FRT-Kan_F	caggcctgtatccggcatgtctgctggttacctgcttccgggaaa atataatgATTCCGGGGATCCGTCGACC	pED208::*tet*$^R\Delta$*traD*
Del-*traD*-FRT-Kan_R	gcccgcatcacccttgatggaagaaatggagagcatcagtattc cctcccgtcatcTGTAGGCTGGAGCTGCTTCG	pED208::*tet*$^R\Delta$*traD*
tet^R-insert_F	cctgtctttactgttgcttcaatcaaacaaaaggaattcacGAA TTCTCATGTTTGACAGCTTATCATCGAT AAGC	To create pED208::*tet*^{R}
tet^R-insert_R	gtttgcctttatcacttccgccagaatctgtcgcagaagcacgTC AGGTCGAGGTGGCCCGGC	To create pED208::*tet*^{R}

*psiB*delchk F	cacaacaacattccagatgcagcctg	pED208Δ*psiB* confirmation
*psiB*delchk_R	gaacgctgatgatactggcctgactg	pED208Δ*psiB* confirmation
*traM*delchk F	ggccgattcacatgtgactattgataatcc	pED208Δ*traM* confirmation
*traM*delchk R	agccggaatcagggaaatgattcc	pED208Δ*traM* confirmation
*oriT*delchk F	gcatcttgccgacagcatattcg	pED208Δ*oriT* confirmation
*oriT*delchk R	gacgcgcaggcctagttcc	pED208Δ*oriT* confirmation
*traD*delchk_F	ataccagacccgcatcgtgtc	pED208Δ*traD* confirmation
*traD*delchk_R	ccggtcttccccgatcagcg	pED208Δ*traD* confirmation
*psiA*delchk_F	gcccgtcactgtcctcagtg	pED208Δ*psiA* confirmation
*psiA*delchk_R	cgggcttcccggcctccacc	pED208Δ*psiA* confirmation
*traI*delchk_F	ggccagcgccgatatggatg	pED208Δ*traI* confirmation
*traI*delchk_R	gtcaggcttatcaccttccgtcc	pED208Δ*traI* confirmation
*ssb*delchk_F	agtccgttc ttccggggag	pED208Δ*ssb* confirmation
*ssb*delchk_R	tgcggggcgt tttcaatgc	pED208Δ*ssb* confirmation
*parA*delchk_F	cgca gaagcagata gctgcttctg	pED208Δ*parA* confirmation
*parA*delchk_R	gcg cagctatatc atggctctgc	pED208Δ*parA* confirmation
*parB1*delchk_F	gc tgctgcaaaa gaagcgatcg	pED208Δ*parB1* confirmation
*parB1*delchk_R	g gaatgccgtg gccatttcg	pED208Δ*parB1* confirmation
*parB2*delchk_F	accgttatat ccgggactac gg	pED208Δ*parB2* confirmation
*parB2*delchk_R	g gatcatggtt ttcagcacat cag	pED208Δ*parB2* confirmation
*cre_Nhe*I_F	agagagGCTAGCaggaggaattcaccatggccaatttactg accgtacacc	pBAD24::*cre*

ssb-strep_F	ccgcagccgttaccggacgattatccgccgatggatgatgatgc cccgttt*TGGTCTCATCCTCAATTCGAAAAGTAA*ATTCCGGGGATCCGTCGACC	Forward primer to fuse Strep at the C-terminal end of SSB
ssb-strep_R	tggtcatgtctttctccttttcctgccccgcaggttgcggggcgt tttcaTGTAGGCTGGAGCTGCTTCG	Reverse primer to fuse Strep at the C-terminal end of SSB
ssb-strep_check_F	gggtgaaggtaacaaccagcg	Forward primer to check Strep fusion at the C-terminal end of SSB
ssb-strep_check_R	agttgatactcctcacagatattgattactg	Reverse primer to check Strep fusion at the C-terminal end of SSB
*cre_Hin*dIII_R	catggtAAGCTTgcatgcctgcaggtcgac	pBAD24::*cre*
Kan_*Bgl*II_F	aacctaAGATCTttagaaaaactcatcgagcatcaaatg	kan^R amplification from pKNT25
Kan_*Nde*I_R	aaacccCATATGagccatattcaacgggaaac	kan^R amplification from pKNT25
BAD33_*NdeI*_F	aacctaCATATGtttagcttccttagctcctg	pBAD33kan^R
BAD33_*Bgl*II_R	aaacccAGATCTtttttttaaggcagttattggtgc	pBAD33kan^R
pcre_F	gcaggcatgcaagcttggc	pAM37, pAM65, pAM39 (*cre* fusions with *psiB*, *psiA*, *traI*)
pcre_*Xba*I_R	agaagaTCTAGAatcgccatcttccagcaggcg	pAM37, pAM65, pAM39 (*cre* fusions with *psiB*, *psiA*, *traI*)
*psiBcre_Xba*I_F	agaagaTCTAGAatgcgtaacattattacccctgatgtgctg	pAM37 (*cre* fusion with *psiB*)
*psiBcre_Hin*dIII_R	aaacccAAGCTTtcataatgtaccctccatagcc	pAM37 (*cre* fusion with *psiB*)
*psiAcre_Xba*I_F	agaagaTCTAGAatgatcccggcaaacctgtcac	pAM65 (*cre* fusion with *psiA*)
*psiAcre_Hin*dIII_R	aaacccAAGCTTtcatgcatggctccggtgtatcag	pAM65 (*cre* fusion with *psiA*)
*ssbcre_Xba*I_F	agaagaTCTAGAatgacagtacgcggcatcaac	pAM87 (*cre* fusion with *ssb*)
*ssbcre_Hin*dIII_R	aaacccAAGCTTtcaaaacggggcatcatcatcc	pAM87 (*cre* fusion with *ssb*)
*parAcre_Xba*I_F	agaagaTCTAGAatgacactgaaaataggacctgtcggc	pAM129 (*cre* fusion with *parA*)
*parAcre_Hin*dIII_R	aaacccAAGCTTggccattacttacctcctgcgagc	pAM129 (*cre* fusion with *parA*)

*parB1cre_Kpn*I_F	agaagaGGTACCatggccaagaaaccgtttatgagacgac	pAM132 (*cre* fusion with *parB1*)
*parB1cre_Sal*I_R	aactatGTCGACtcactgagagcgcatccctttgac	pAM132 (*cre* fusion with *parB1*)
*parB2cre_Xba*I_F	agaagaTCTAGAatgacactgaaaataggacctgtcggc	pAM89 (*cre* fusion with *parB2*)
*parB2cre_Hin*dIII_R	aaacccAAGCTTtcaggctgcatctggaatg	pAM89 (*cre* fusion with *parB2*)
*traIcre_Xba*I_F	agaagaTCTAGAatgctctccatttcttccatcaaggg	pAM69 (*cre* fusion with *traI*)
*traIcre_Hin*dIII_R	aaacccAAGCTTctgaatcagtccccaccgagc	pAM69 (*cre* fusion with *traI*)
*traI*cloning_*Nhe*I_F	aacctaGCTAGCaggaggaattcaccatgctctccatttcttc catcaaggg	pAM69 (pBAD24::*traI*)
*traI*cloning_*Hin*dIII_R	aaacccAAGCTTctgaatcagtccccaccgagc	pAM69 (pBAD24::*traI*)
*traM*cloning_*Nhe*I_F	aacctaGCTAGCaggaggaattcaccatgccaaaaataca gacttatgtgaacaataatgtttatg	pAM53 (pBAD24::*traM*)
*traM*cloning_*Hin*dIII_R	aaacccAAGCTTtcactcctgatcatcctcatcatcagg	pAM53 (pBAD24::*traM*)
*psiB*cloning_*Nhe*I_F	aacctaGCTAGCaggaggaattcaccatgcgtaacattatta cccctgatgtgc	pAM93 (pBAD24::*psiB*)
*psiB*cloning_*Hin*dIII_R	aaacccAAGCTTtcataatgtaccctccatagcc	pAM93 (pBAD24::*psiB*)
*psiA*cloning_*Nhe*I_F	aacctaGCTAGCaggaggaattcaccatgatcccggcaaa cctgtcac	pAM66 (pBAD24::*psiB*)
*psiA*cloning_*Hin*dIII_R	aaacccAAGCTTtcatgcatggctccggtgtatcag	pAM66 (pBAD24::*psiB*)
*ssb*cloning_*Nhe*I_F	aacctaGCTAGCaggaggaattcaccatggcagttcgtggc attaacaaggtc	pAM98 (pBAD24::*ssb*)
*ssb*cloning_*Hin*dIII_R	aaacccAAGCTTtcagaacgggatatcgtctgaaaacccg	pAM98 (pBAD24::*ssb*)
*oriT*cloning_*Kpn*I_F	aacctaGGTACCttgagcgcatt atcacgc	pAM118 (pBAD33::$oriT_{pED208}$)
*oriT*cloning_*Hin*dIII_R	aaacccAAGCTTtgttcacataagtctgtatttttgg	pAM118 (pBAD33::$oriT_{pED208}$)
*traD*cloning_*Kpn*I_F	aaactaGGTACCaggaggaattcaccatgagcctgaatcct cgcg	pAM110 (pBAD24kan^R::*traD*)
*traD*cloning_*Hin*dIII_R	agagagAAGCTTtcagtattccctcccgtcatccatctc	pAM110 (pBAD24kan^R::*traD*)
*traD*ΔC15cloning_*Kpn*I_F	aaactaGGTACCaggaggaattcaccatgagcctgaatcct cgcg	pAM112 (pBAD24kan^R::*traD*ΔC15)
*traD*ΔC15cloning_*Hin*dIII_R	aacctaAAGCTTtcagttaatgttgacctcttccctgcg	pAM112 (pBAD24kan^R::*traD*ΔC15)
ssbRT_F	CGATAAACAGACCGGAGAGATG	Forward primer to amplify *ssb* by RT-PCR
ssbRT_R	GATACTCACTGGCCACTTCTG	Reverse primer to amplify *ssb* by RT-PCR
psiBRT_F	TGATGCGTGACCTTGACTG	Forward primer to amplify *psiB* by RT-PCR

psiBRT_R	GATGGCGTGAAACGGATCT	Reverse primer to amplify *psiB* by RT-PCR
psiART_F	ATCGACCGGCTGATTGAAAG	Forward primer to amplify *psiA* by RT-PCR
psiART_R	GTTCGCTCCTGCATCTGAAA	Reverse primer to amplify *psiA* by RT-PCR
16SrRNART_F	ACGGCCGCAAGGTTAAA	Forward primer to amplify 16S rRNA by RT-PCR
16SrRNART_R	GTGGATGTCAAGACCAGGTAAG	Reverse primer to amplify 16S rRNA by RT-PCR
gyrART_F	TCAGCGGAGAACAGCATTAC	Forward primer to amplify *gyrA* by RT-PCR
gyrART_R	CCGGTAAAGTGGCGATCAA	Reverse primer to amplify *gyrA* by RT-PCR

[a] For construction of deletion mutations on pED208, the sequences specific to pKD3 or pKD13 are in uppercase. For construction of the *ssb-strep* insertion mutant, the sequence specific to *strep* is in uppercase and italic. For plasmid constructions, restriction sites used for cloning are in uppercase and underlined.
