# Supplementary material for: Protein Transfer through an F Plasmid-Encoded Type IV Secretion System Suppresses the Mating-Induced SOS Response
Source: mBio. 2021 Jul 13;12(4):e01629-21. doi: 10.1128/mBio.01629-21 (PMC8406263; doi:10.1128/mBio.01629-21)
Supplement: TABLE S1 [file mbio.01629-21-st001.pdf]

Table S1. Strains and plasmids used in this study

Strains	Description	Source or Reference
HME45	W3110 Δ*lacU169* λ*c*1857 Δ(*cro-bioA*) (carries λ red recombination functions) F^-	(1)
MC4100	*araD139* λ^- *e14*$^-$ *Δ(argF-lac)169 rpsL150*(strR) *flhD5301 Δ(fimB-fimE)632* F^-	(2)
JCM158	MC4100 *ara*$^{R/-}$; spontaneous arabinose resistant	(3)
MC4100 *yaeH*	MC4100 Δ*yaeH*::*cat*	Anna Konovalova
AA116	MC4100 Δ*yaeH*::*cat*, Rif^r	This study
AA712	MC4100 Nal^R	This study
CSH26	*ara* Δ(*lac-pro*) *thi*	(4)
CSH26Cm::LTL	CSH26 *galK*::*cat*::*loxP*-*tet*^{R}-*loxP*	(5)
DH5α	*endA1 glnV44 thi-1* *recA1 relA1 gyrA96 deoR nupG purB20* φ80d*lacZ*ΔM15 Δ(*lacZYA-argF*)U169, hsdR17($r_K^- m_K^+$), λ^- F^-	(6)
MG1655	*rph-1* λ F^-	(7)
SMR17964	MG1655 Δ*att*λ::P_{sulA}*mCherry*	(8)
SMR17962	MG1655 Δ*att*λ::P_{sulA}*mCherry* FRT*cat*FRT	(8)
SMR17966	MG1655 Δ*att*λ::P_{sulA}*mCherry* FRT *lexA3*(Ind$^-$) *malB*::Tn*9*	(9)
SMR21400	MG1655 Δ*att*λ::P_{sulA}*mCherry* FRT Δ*sulA*::FRT *recG258*::Tn*10*Kan	(9)
**Plasmid**	**Genotype or description**	**Source or Reference**
pED208	A natural derivative of Fo-*lac*$^+$ IncFV	(10)
pBBR1MCS	*tet*^{R} gene bearing plasmid	(11)
pED208::*tet*^{R}	pED208 bearing *tet*^{R}	This study
pED208::*spc*^{R}	pED208 bearing *spc*^{R}	(12)
pED208::*spc*^{R}Δ*traA*	pED208::*spc*^{R} deleted of *traA*	(12)
pED208:: *spc*^{R}Δ*traD*	pED208::*spc*^{R} deleted of *traD*	(12)
pED208::*tet*^{R}Δ*traC*	pED208::*tet*^{R} deleted of *traC*	(12)
pED208::*spc*^{R}Δ*traI*	pED208::*spc*^{R} deleted of *traI*	This study
pED208::*spc*^{R}Δ*psiB*	pED208::*spc*^{R} deleted of *psiB*	This study
pED208::*spc*^{R}Δ*psiA*	pED208::*spc*^{R} deleted of *psiA*	This study
pED208::*spc*^{R}Δ*ssb*	pED208::*spc*^{R} deleted of *ssb*	This study
pED208::*spc*^{R}Δ*parA*	pED208::*spc*^{R} deleted of *parA*	This study
pED208::*spc*^{R}Δ*parB1*	pED208::*spc*^{R} deleted of *parB1*	This study
pED208::*spc*^{R}Δ*parB2*	pED208::*spc*^{R} deleted of *parB2*	This study

pED208::*spc*^{R}Δ*traM*	pED208::*spc*^{R} deleted of *traM*	This study
pED208::*spc*^{R}Δ*oriT*	pED208::*spc*^{R} deleted of *oriT* sequence	This study
pED208::*tet*^{R}Δ*traD*	pED208::*tet*^{R} deleted of *traD*	This study
pED208::*ssb-str*	pED208::spcR with strep-tagged *ssb* substituted for *ssb*	This study
pBAD24	Crb^R; ColE1 plasmid with arabinose-inducible P_{BAD} promoter	(13)
pBAD33	Chl^R; pACYC184 plasmid with arabinose-inducible P_{BAD} promoter	(13)
pKNT25	BATCH two-hybrid vector, used for source of *kan*^{R} gene	(14)
pTB33	pBAD33 carrying *cre*	(15)
pKD13	Crb^R, R6Kγ plasmid with FRT*kan*^{R}FRT	(16)
pKD3	Crb^R, R6Kγ plasmid with FRT*cat*FRT	(16)
pCP20	Yeast Flp recombinase on a temperature-sensitive replicon λ*cIts857;* Crb^R, Chl^R	(16)
pPK18	pBAD24 with P_{BAD}::*traD*	(12)
pAM108	pBK33; Chl^R gene in pBAD33 was replaced with Kan^R gene from pKNT25	This study
pAM38	pBAD24 with P_{BAD}::*cre*	This study
pAM37	pBAD24 with P_{BAD}::*cre-psiB*	This study
pAM65	pBAD24 with P_{BAD}::*cre-psiA*	This study
pAM39	pBAD24 with P_{BAD}::*cre-traI*	This study
pAM69	pBAD24 with P_{BAD}::*traI*	This study
pAM53	pBAD24 with P_{BAD}::*traM*	This study
pAM93	pBAD24 with P_{BAD}::*psiB*	This study
pAM66	pBAD24 with P_{BAD}::*psiA*	This study
pAM118; p$oriT_{pED208}$	pBAD33 with *oriT* sequence of pED208	This study
pAM110	pBAD33*kan*^{R} with P_{BAD}::*traD*	This study
pAM112	pBAD33*kan*^{R} with P_{BAD}::*traD*ΔC15	This study
pAM87	pBAD24 with P_{BAD}::*cre-ssb*	This study
pAM129	pBAD24 with P_{BAD}::*cre-parA*	This study
pAM132	pBAD24 with P_{BAD}::*cre-parB1*	This study
pAM89	pBAD24 with P_{BAD}::*cre-parB2*	This study
pAM98	pBAD24 with P_{BAD}::*ssb*	This study

References

1. **Yu D, Ellis HM, Lee EC, Jenkins NA, Copeland NG, Court DL.** 2000. *Proc Natl Acad Sci U S A* **97:**5978-5983. doi:10.1073/pnas.100127597
2. **Casadaban MJ.** 1976. *J Mol Biol* **104:**541-555. doi:10.1016/0022-2836(76)90119-4
3. **Wu T, Malinverni J, Ruiz N, Kim S, Silhavy TJ, Kahne D. 2005.** *Cell* **121:**235-245. doi: 10.1016/j.cell.2005.02.015
4. **Miller JH.** 1972. Experiments in Molecular Genetics. Cold Spring Harbor Laboratory, Cold Spring Harbor, N.Y.

5. **Lang S, Gruber K, Mihajlovic S, Arnold R, Gruber CJ, Steinlechner S, Jehl MA, Rattei T, Frohlich KU, Zechner EL.** 2010. *Mol Microbiol* **78:**1539-1555. doi:10.1111/j.1365-2958.2010.07423.x
6. **Grant SG, Jessee J, Bloom FR, Hanahan D.** 1990. Differential plasmid rescue from transgenic mouse DNAs into *Escherichia coli* methylation-restriction mutants. *Proc Natl Acad Sci U S A* **87:**4645-4649. doi:10.1073/pnas.87.12.4645
7. **Guyer MS, Reed RR, Steitz JA, Low KB.** 1981. *Cold Spring Harb Symp Quant Biol* 45 Pt 1:135-140. doi:10.1101/sqb.1981.045.01.022
8. **Nehring RB, Gu F, Lin HY, Gibson JL, Blythe MJ, Wilson R, Bravo Nunez MA, Hastings PJ, Louis EJ, Frisch RL, Hu JC, Rosenberg SM.** 2016. *Nucleic Acids Res* **44:**e41. doi:10.1093/nar/gkv1131
9. **Xia J, Chiu LY, Nehring RB, Bravo Nunez MA, Mei Q, Perez M, Zhai Y, Fitzgerald DM, Pribis JP, Wang Y, Hu CW, Powell RT, LaBonte SA, Jalali A, Matadamas Guzman ML, Lentzsch AM, Szafran AT, Joshi MC, Richters M, Gibson JL, Frisch RL, Hastings PJ, Bates D, Queitsch C, Hilsenbeck SG, Coarfa C, Hu JC, Siegele DA, Scott KL, Liang H, Mancini MA, Herman C, Miller KM, Rosenberg SM.** 2019. *Cell* **176:**127-143 e24. doi:10.1016/j.cell.2018.12.008
10. **Falkow S, Baron LS.** 1962. *J Bacteriol* **84:**581-9. doi:10.1128/JB.84.3.581-589.1962
11. **Kovach ME, Phillips RW, Elzer PH, Roop RM, 2nd, Peterson KM.** 1994. *Biotechnique*s **16:**800-802.
12. **Hu B, Khara P, Christie PJ. 2019.** *Proc Natl Acad Sci U S A* **116:**14222-14227. doi:10.1073/pnas.1904428116
13. **Guzman LM, Belin D, Carson MJ, Beckwith J.** 1995. *J Bacteriol* **177:**4121-30. doi:10.1128/jb.177.14.4121-4130.1995
14. **Karimova G, Dautin N, Ladant D. 2005**. *J Bacteriol* **187:**2233-43. doi:10.1128/JB.187.7.2233-2243.2005
15. **Whitaker N, Berry TM, Rosenthal N, Gordon JE, Gonzalez-Rivera C, Sheehan KB, Truchan HK, VieBrock L, Newton IL, Carlyon JA, Christie PJ. 2016.** *J Bacteriol* **198:**2701-18. doi:10.1128/JB.00378-16
16. **Datsenko KA, Wanner BL. 2000.** *Proc Natl Acad Sci U S A* **97:**6640-6645. doi:10.1073/pnas.120163297
